# The wild sweetpotato (*Ipomoea trifida*) genome provides insights into storage root development

**DOI:** 10.1186/s12870-019-1708-z

**Published:** 2019-04-01

**Authors:** Ming Li, Songtao Yang, Wei Xu, Zhigang Pu, Junyan Feng, Zhangying Wang, Cong Zhang, Meifang Peng, Chunguang Du, Feng Lin, Changhe Wei, Shuai Qiao, Hongda Zou, Lei Zhang, Yan Li, Huan Yang, Anzhong Liao, Wei Song, Zhongren Zhang, Ji Li, Kai Wang, Yizheng Zhang, Honghui Lin, Jinbo Zhang, Wenfang Tan

**Affiliations:** 1 Institute of Biotechnology and Nuclear Technology, Sichuan Academy of Agricultural Sciences, Chengdu, 610061 Sichuan People’s Republic of China; 2 Crop Research Institute, Sichuan Academy of Agricultural Sciences, Chengdu, 610066 Sichuan People’s Republic of China; 3 Key Laboratory of Bio-Resource and Eco-Environment of Ministry of Education, College of Life Sciences, Sichuan University, Chengdu, 610065 Sichuan People’s Republic of China; 4 Novogene Bioinformatics Institute, Beijing, 100083 People’s Republic of China; 5 Guangdong Provincial Key Laboratory of Crops Genetics and Improvement, Crops Research Institute, Guangdong Academy of Agricultural Sciences, Guangzhou, 510640 Guangdong People’s Republic of China; 6 Department of Biology, Montclair State University, Montclair, NJ 07043 USA

**Keywords:** *Ipomoea trifida* genome, Sweetpotato, Evolution, Storage root development, QTL, *BMY11* (*beta-amylase*)

## Abstract

**Background:**

Sweetpotato (*Ipomoea batatas* (L.) Lam.) is the seventh most important crop in the world and is mainly cultivated for its underground storage root (SR). The genetic studies of this species have been hindered by a lack of high-quality reference sequence due to its complex genome structure. Diploid *Ipomoea trifida* is the closest relative and putative progenitor of sweetpotato, which is considered a model species for sweetpotato, including genetic, cytological, and physiological analyses.

**Results:**

Here, we generated the chromosome-scale genome sequence of SR-forming diploid *I. trifida* var. Y22 with high heterozygosity (2.20%). Although the chromosome-based synteny analysis revealed that the *I. trifida* shared conserved karyotype with *Ipomoea nil* after the separation, *I. trifida* had a much smaller genome than *I. nil* due to more efficient eliminations of LTR-retrotransposons and lack of species-specific amplification bursts of LTR-RTs. A comparison with four non-SR-forming species showed that the evolution of the beta-amylase gene family may be related to SR formation. We further investigated the relationship of the key gene *BMY11* (with identity 47.12% to *beta-amylase 1*) with this important agronomic trait by both gene expression profiling and quantitative trait locus (QTL) mapping. And combining SR morphology and structure, gene expression profiling and qPCR results, we deduced that the products of the activity of *BMY11* in splitting starch granules and be recycled to synthesize larger granules, contributing to starch accumulation and SR swelling. Moreover, we found the expression pattern of *BMY11*, sporamin proteins and the key genes involved in carbohydrate metabolism and stele lignification were similar to that of sweetpotato during the SR development.

**Conclusions:**

We constructed the high-quality genome reference of the highly heterozygous *I. trifida* through a combined approach and this genome enables a better resolution of the genomics feature and genome evolutions of this species. Sweetpotato SR development genes can be identified in *I. trifida* and these genes perform similar functions and patterns, showed that the diploid *I. trifida* var. Y22 with typical SR could be considered an ideal model for the studies of sweetpotato SR development.

**Electronic supplementary material:**

The online version of this article (10.1186/s12870-019-1708-z) contains supplementary material, which is available to authorized users.

## Background

Sweetpotato (*Ipomoea batatas* (L.) Lam.), which is mainly cultivated for its underground storage root (SR), was found in the Americas approximately 8000–10,000 years ago and domesticated at least 4000 years ago [1]. This plant was then spread around the world, which could be traced back to the beginning of pre-Columbian times [2]. Because it is easy to grow, with high yield and an abundance of starch and nutrients, it has become an important part of diets around the world. In recent years, the global production of sweetpotato was more than 100 million tons (http://www.fao.org/faostat/en/#home); it has become one of the three major root and tuber crops and the seventh most important food crop in the world [3–5]. Understanding the mechanism of SR formation and development is of pivotal importance for further improving sweetpotato yield [6]. To date, sporamin proteins, Dof-type zinc finger proteins, MADS-box proteins and KNOX proteins have been shown to be associated with SR development [7–9]. The lack of genomic information has slowed research into SR development [7]. The candidate genes corresponding to many sweetpotato quantitative trait loci (QTL) remain elusive [10]. The newly released haplotype sweetpotato genome [11] provides an additional resource to help reach this goal, but the chromosome-scale assembly was performed according to the *Ipomoea nil* genome, which might not be suitable for genomics research, such as QTL investigations. Sweetpotato has a large number of small chromosomes (2*n* = 6*x* = 90, B_1_B_1_B_2_B_2_B_2_B_2_), with highly repetitive elements and high heterozygosity, and it contains a homogenous B_2_subgenome [11, 12], which causes difficulty in generating high-quality sequences at the chromosome level [13].

Among approximately 700 species of the genus *Ipomoea* [14], *I. trifida* is the closest wild relative of sweetpotato [12, 15, 16]. Artificial crossing and cytogenetic research suggest that hexaploid *I. trifida* arose from diploid *I. leucantha* and tetraploid *I. littoralis*, while tetraploid *I. littoralis* was an autopolyploid from the *I. leucantha* B genome [17, 18]*. I. leucantha* and *I. littoralis* should be considered the diploid and tetraploid forms of *I. trifida* based on interspecific hybridization and cytogenetics, respectively [18, 19]. The artificial hexaploid *I. trifida* produced from diploid and tetraploid *I. trifida* has the same chromosome types as sweetpotato, and thus, sweetpotato may derive from hexaploid *I. trifida* [18–20]. Moreover, data from noncoding chloroplast and nuclear ITS sequences and nuclear SSRs supported an autopolyploid origin of sweetpotato from a progenitor that shared the diploid *I. trifida* genome [16]. Triploid *I. trifida* may have provided a bridge from diploid and tetraploid to hexaploid *I. trifida* [16, 19, 20]. Our recent result of restriction-site-associated DNA sequencing (RAD-seq) indicated that sweetpotato originated from hexaploid *I. trifida*, and that hexaploid *I. trifida* evolved from tetraploid *I. trifida* and diploid *I. trifida* [21]. However, *Wx* intron variations support an allohexaploid origin of sweetpotato from *I. tenuissima* and tetraploid *I. littoralis* Blume or tetraploid *I. tabascana*, and the *I. tenuissima* derived earlier from diploid *I. trifida* and an unidentified species [22]. Besides, the results of the newly released haplotype-resolved sweetpotato genome also suggested that sweetpotato may the result of an initial cross between a tetraploid progenitor and a diploid *I. trifida* progenitor [11]. Either it is the most likely progenitor of sweetpotato or a part of its genome has introgressed into that of sweetpotato [23]. In any case, diploid *I. trifida* is at a critical point in the complex evolutionary history of sweetpotato, and the origin of sweetpotato remains disputed. Therefore, many studies have focused on diploid *I. trifida*; a genome survey has been reported [24], and another genome assembly can be viewed online (http://sweetpotato.plantbiology.msu.edu/new.shtml). Previous studies have mainly focused on the fibrous root (FR), pencil root (PR) or thick root (TR) of *I. trifida* [20, 24–28]. Few studies have reported on the SR, possibly because of a lack of diploid material with SR, and use of the genome to study SR development has not yet been reported.

Here, we report a high-quality, chromosome-anchored reference genome of the diploid *I. trifida* var. Y22 [21], which has typical SR and is similar to sweetpotato (Additional file 1: Figure S1). We de novo assembled the highly heterozygous genome (2.20%) with a combined strategy, and 30,227 gene models were predicted. We found that a whole-genome triplication (WGT) occurred before its speciation, approximately 74.5 million years ago (MYA). Through an integrated analysis of gene family evolution, root comparative transcriptomes, QTL mapping, qPCR, we found that the new gene *BMY11* (with identity 47.12% to *beta-amylase 1*) may play a key role in the process of SR development. The key genes between Y22 and sweetpotato have a similar expression pattern, and sweetpotato SR development genes can be identified by QTL mapping of the diploid *I. trifida* genetic population. This work will be very helpful to further understand the complex evolutionary history and SR development mechanisms of *I. trifida* and sweetpotato.

## Results

### Genome assembly, validation and annotation

We constructed a 180 bp paired-end (PE) library and sequenced the Y22 genome using Illumina PE125, which yielded 62.27 Gb of data for genome survey analysis. K-mer frequency [29] distribution analysis showed that the genome of Y22 was 476.6 Mb, consistent with the estimate based on flow cytometry [30], and the heterozygosity and the proportion of repeat sequences were 2.20 and 48.42%, respectively (Additional file 2: Table S1, Additional file 1: Figures S2 and S3). To achieve a high-quality and chromosome-scale reference genome, 125.6 Gb of clean Illumina shotgun reads (approximately 264-fold coverage of the genome) and 537 Mb of Illumina Moleculo synthetic long reads (approximately 1-fold coverage of the genome) (Additional file 2: Table S2) were initially assembled into 431.57 Mb of sequence with a contig N50 of 26.50 Kb and a scaffold N50 of 580.68 Kb (Additional file 2: Table S3). Then, we incorporated 10.05 Gb (21-fold coverage of the assembly) of PacBio RS II data with an N50 read length of 17.03 Kb to increase sequence continuity. The contig and scaffold N50 of the final assembly were 54.49 Kb and 607.92 Kb, respectively (Additional file 2: Table S4). Additionally, 96.71% of the *I. trifida* genome was covered by the assembled 460.93 Mb scaffolds. A genotype-by-sequencing (GBS) [31, 32] genetic map was constructed using 200 true F1 individuals (Y25 × Y22) (Additional file 1: Figures S1 and S4; Additional file 2: Tables S5 and S6), and 400.44 Mb of sequence (86.88% of the final scaffolds) was successfully anchored to 15 chromosomes (Additional file 1: Figures S5 and S6), which could be considered as a better reference for *I. trifida* than the recent released genome (373.4 Mb scaffolds were anchored to 15 linkage groups) [28].

The quality and completeness of the assembly were evaluated by various datasets. First, we mapped the short insert size library reads to the assembled genome; the mapping rate was 95.2, and 95.76% of the assembled genome had more than 20-fold coverage (Additional file 2: Table S7). The GC content and sequence depth distribution calculated for the 10 k non-overlapping sliding window of the assembled genome showed that the assembled genome was not contaminated (Additional file 1: Figures S7 and S8). Second, the full-length transcripts assembled from 7.2 Gb of leaf RNA-seq reads by Trinity [33] were aligned back to the assembled genome. The results showed that more than 98.80% of the full-length transcript sequences could be mapped back onto one scaffold with sequence coverage more than 50% (Additional file 2: Table S8), which was comparable to the results of the highly heterozygous assembly [34]. Third, 98.39% of 248 core eukaryotic genes (Cluster of Essential Genes (CEG) database) [35] could be aligned back to the genome assembly with high confidence (Additional file 2: Table S9). And the 93.9% of the BUSCO [36] conserved genes were complete in the assembly. These results indicated that our assembled genome achieved complete coverage of the conserved genes.

**Fig. 1:**
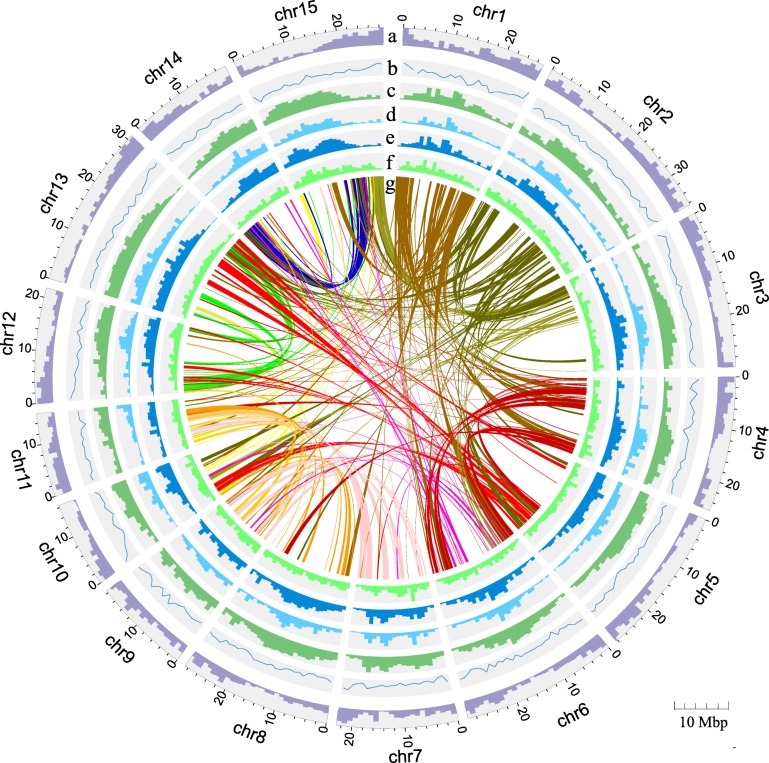
Genome characterization of diploid *I. trifida*. **a** Gene density per Mb. **b** GC content per Mb. **c** Repeat content per Mb. **d** *Gypsy* content per Mb. **e** *Copia* content per Mb. **f** DNA repeat content per Mb. **g** The syntenic regions between different chromosomes were identified by MCScanX. Syntenic regions larger than 350 Kb were shown. Each chromosome is assigned a color, and the color of the links between chromosomes is determined by the color of chromosome which has a smaller number in the pair

In total, 30,227 gene models were predicted in the assembled genome of *I. trifida* and 79.76% of these genes were supported by expression evidence (RNA-seq reads) from seven different tissues including leaf, flower, stigma, pollen, stem, root and seed (Additional file 2: Table S10). Additionally, 84.75% of all the gene models had homology hits with > 50% high-scoring segment pair coverage in the sequences of seven species, including *Arabidopsis thaliana*, *Beta vulgaris*, *Capsicum annuum*, *Sesamum indicum*, *Solanum lycopersicum*, *Solanum tuberosum* and *Vitis vinifera* (Additional file 1: Figure S9). A total of 28,456 genes (94.14% of all genes) were annotated based on homology to known proteins from the Kyoto Encyclopedia of Genes and Genomes (KEGG), Swiss-Prot, TrEMBL and Gene Ontology (GO) public databases (Additional file 2: Table S11). We identified the candidate noncoding RNA (ncRNA) sequences for *I. trifida* by comparison with known ncRNA libraries and by structural prediction, and the ratio of miRNA and tRNA in the genome were identified as 0.0310 and 0.0423%, respectively (Additional file 2: Table S12). There were 50.86% repeat sequences in the genome, including 5.92% tandem repeats. Long terminal repeat (LTR) retrotransposons were the most abundant elements, comprising 30.41% of the genome, whereas DNA transposons, long interspersed nuclear elements (LINEs), and short interspersed nuclear elements (SINEs) accounted for 13.15, 4.86 and 0.68% of the genome, respectively (Additional file 2: Table S13). We mapped the distributions of genes, GC contents, repetitive sequences, *Gypsy*, *Copia*, and DNA repeats of the *I. trifida* genome to obtain an overview of the genome characterization (Fig. 1). We found that the transposable elements are primarily located within chromosome pericentromeric regions, while most genes are located on the chromosome arms.

### Comparative genomics analysis

**Fig. 2:**
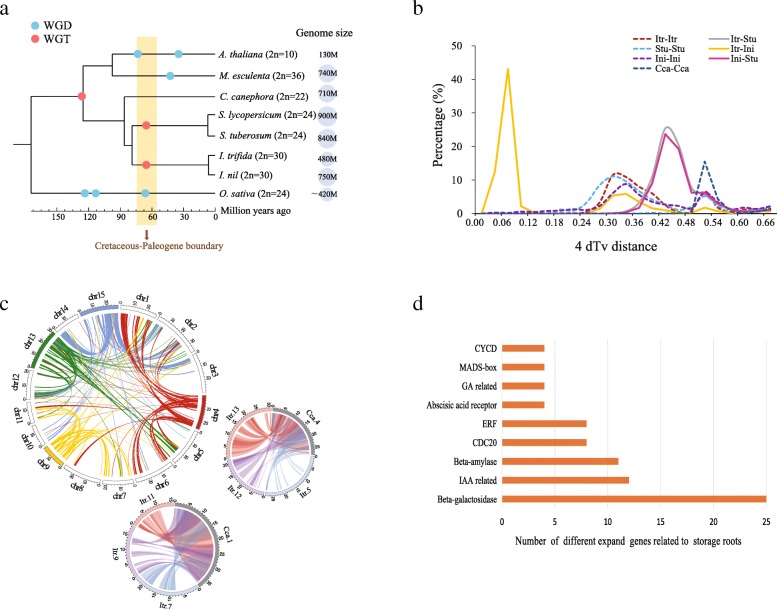
Evolution of the *I. trifida* genome and gene families. **a** Phylogenetic tree showing the divergence times of eight species. The blue dots represent WGD events, and the red dots represent WGT events. The grey circles indicate the genome sizes of the eight species. **b** Distribution of 4dTv values of syntenic genes. The dotted lines represent comparisons of each species with its own sequences, and peaks indicate genome duplication/triplication events. The solid peaks indicate divergence events between species. Itr: *I. trifida*, Stu: *S. tuberosum*, Cca: *C. canephora*, Ini: *I. nil*. **c** The large circle shows *I. trifida* - *I. trifida* intragenomic syntenic regions putatively derived from WGT, which were detected by a series of paralogous genes (only chromosomes 4, 9, 13 and 15 are shown). The bottom circle shows collinear blocks between chromosome 1 of *C. canephora* and chromosomes 6, 7 and 11 of *I. trifida*. The right circle shows collinear blocks between chromosome 4 of *C. canephora* and chromosomes 5, 12 and 13 of *I. trifida*. The colour of each link corresponds to the colour of the chromosome. **d** The expansion of gene families associated with SR in *I. trifida*

Phylogenetic inference from eight plant genomes (Additional file 2: Table S14) using 1930 single-copy gene families illustrated that *I. trifida* and *I. nil* have a common ancestor, which separated from a common ancestor of *S. tuberosum* and *Ipomoea* spp. approximately 78.8 MYA and *I. trifida* and *I. nil* diverged from their common ancestor around 6.4 MYA (Fig. 2a). Despite their close relationship, the estimated genome size of *I. trifida* was much smaller than that of *I. nil* (476.6 Mb vs 750 Mb) [37]. A comparison of the repeat contents of these two Convolvulaceae genomes showed that the *I. nil* genome contains a higher abundance of repetitive elements, especially the LTR retrotransposon family, than that of *I. trifida* does (Additional file 2: Table S15). The solo LTR / intact LTR ratio of *I. trifida* (0.96:1) is notably higher than that of *I. nil* (0.38:1), indicating higher recombination frequencies in the *I. trifida* genome, which may eliminate transposable elements (Additional file 2: Table S16). Furthermore, we identified collinear genome regions between *I. trifida* and *I. nil* based on the gene blocks detected by MCScanX [38]. The overall size of the syntenic blocks in the *I. trifida* genome is 248.3 Mb, which is smaller than that in the *I. nil* genome (339.9 Mb). In the syntenic blocks, the total length of the repetitive sequences of *I. trifida* is 97.1 Mb, less than that of *I. nil* (165.0 Mb). The repeat sequences occupied 39.1% of these syntenic blocks in *I. trifida*, a smaller ratio than that of *I. nil* (48.5%) (Additional file 2: Table S17). These results suggest that repetitive elements are the major factors contributing to the difference in genome size between *I. trifida* and *I. nil*, probably due to the species-specific amplification bursts of LTR-RTs in *I. nil* (Additional file 1: Figure S10) and more efficient eliminations of LTR-retrotransposons in *I. trifida*. Although the transposable elements of *I. nil* are more prevalent than those of *I. trifida*, the two species share the same chromosome number, indicating that no large-scale chromosome fission or fusion occurred after their speciation (Additional file 1: Figure S11). The newly released haplotype-resolved sweetpotato assembly has similar chromosome synteny with *I. trifida* (Additional file 1: Figure S12), which is as expected, because the sweetpotato pseudochromosomes were generated according to gene and sequence synteny between sweetpotato and *I. nil* [11]. Although Yang et al. has developed a novel haplotyping method to efficiently solve the assembly problem for polyploidy genomes, the monoploid assembly has a lower scaffold N50 (~ 201 Kb) due to its high polymorphism level and limited sequencing depth (~ 67-fold based on hexaploid genome size). Hence, more detailed comparisons between *I. trifida* and sweetpotato should be carried out once a more high-quality chromosome reference of the sweetpotato genome is obtained with the integration of long-read sequencing technology, Hi-C sequencing and assembly algorithms developed for polyploid genomes [39].

Whole-genome duplication (WGD) events in *I. trifida* were investigated using paralogous regions detected by protein sequence similarity to reveal their importance in genome evolution [40]. We identified 1856 intra diploid *I. trifida* syntenic blocks (with at least 5 genes per block), which contained 17,630 genes, accounting for ~ 58.33% of the total genes. A four-fold transversion (4dTv) analysis indicated that the WGD occurred at the peak around 0.31, approximately 74.5 MYA (Fig. 2a, b) which is similar to the reported result [37]. Notably, these WGD/WGT events of this historical period may have enabled the survival of the most recent common ancestor of these species across the Cretaceous-Paleogene boundary (Fig. 2a), as attested in many other angiosperms [41]. A gene block comparison of *I. trifida* versus itself also showed numerous duplicated gene pairs (Fig. 1), which is clear structural evidence of a WGT event*.* We found that chromosomes 4, 9, 13 and 15 of *I. trifida* each obviously have two additional paralogous segments from other chromosomes (Fig. 2c). This result indicated that a WGT event occurred in the *Ipomoea* genome instead of the reported WGD [37]. To confirm this WGT event, we further compared the genome of *I. trifida* with that of *Coffea canephora*, which has no lineage-specific WGD/WGT event except the older *gamma* genome triplication in the common ancestor of most eudicots [42]. As expected, the structural analysis showed that one *C. canephora* region tends to correspond to three segments of *I. trifida.* More specifically, chromosome 1 of *C. canephora* has a syntenic relationship with chromosomes 7, 9 and 11 of *I. trifida*, and chromosome 4 of *C. canephora* has a syntenic relationship with chromosomes 5, 12 and 13 of *I. trifida* (Fig. 2c)*.* Furthermore, the mutual coverage of *C. canephora* and *I. trifida* achieved the maximal value under a corresponding relationship of 1:3. Similarly, comparing *I. trifida* with *Vitis vinifera*, the genome coverage reached a maximal value of 93.2% for *I. trifida* and of 95.8% for *V. vinifera* at the ratio of 3:1, which also supports the results of the above WGT analysis (Additional file 2: Table S18).

The expanded and contracted gene families of *I. trifida* were identified using CAFE [43] among *I. trifida* and four non-SR-forming species, including *A. thaliana*, *S. lycopersicum*, *C. canephora* and *I. nil.* There were 910 expanded gene families enriched in 22 pathways (*p* value< 0.05). Notably, some of these families have been reported to have functions in carbohydrate metabolism, for example, glycosaminoglycan degradation, carbon metabolism, and carbon fixation in photosynthetic organisms (Additional file 2: Table S19). Among these expanded gene families, we found that several of them may be important for SR development based on the functional annotation, such as *beta-amylase* genes [3, 44], *MADS-box* genes [45, 46], ethylene-responsive transcription factor (*ERF*) genes [47], *beta-galactosidase* genes [48], IAA-related genes [49, 50] and gibberellic acid (GA)-related genes such as *GA20ox* and gibberellin-regulated protein [51] (Fig. 2d). Besides, there were 100 contracted gene families enriched in 15 KEGG pathways, such as plant-pathogen interaction, ABC transporters, and ascorbate and aldarate metabolism (Additional file 2: Table S20).

### Identification of key genes in SR development

**Fig. 3:**
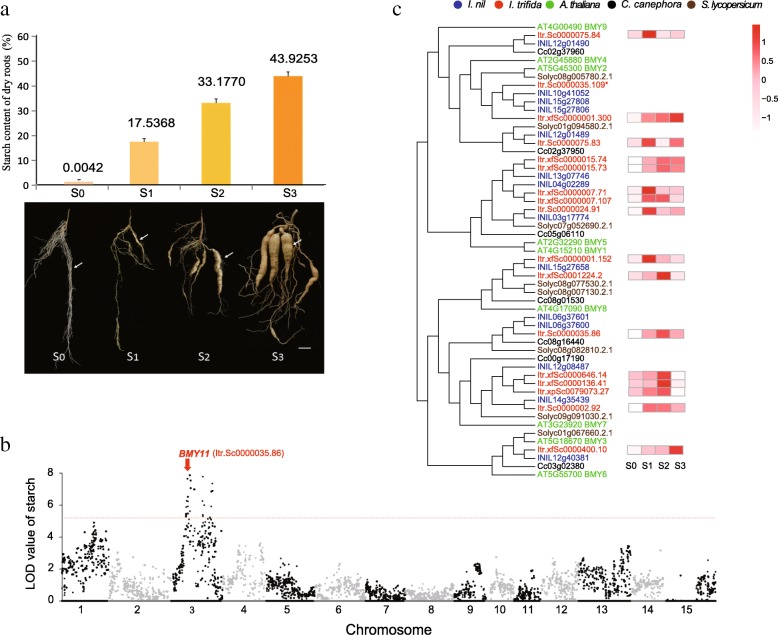
Identification of *BMY11*. **a** The graph above shows the starch contents of four typical stages of SR development. The image below shows the four typical stages (white arrow) of SR development. S0, adventitious root (AR); S1, initiating storage root (ISR); S2, young storage root (YSR); S3, mature storage root (MSR). Bar = 2 cm. **b** *BMY11* (*Itr.Sc0000035.86*) was located on chromosome 3. **c** Phylogenetic tree of the *beta-amylase* genes from five species. Different colours represent different species. The right side shows expression heatmaps of the 17 *beta-amylase* genes of *I. trifida*. * The heatmaps of genes with RPKM = 0 were not shown due to invalid calculation

We tested the root diameters and starch contents of four typical stages in *I. trifida* SR development. The starch content of adventitious root (AR, S0) was 0.0042%; it rapidly increased to 17.54% in initiating storage root (ISR, S1) and reached 43.93% in mature storage root (MSR, S3) (Fig. 3a). The root diameter increased from that of AR to more than 20 mm (Additional file 1: Figure S1). These results indicated that *I. trifida* SR development is strongly associated with starch accumulation. We thus compared gene expression using the RNA-seq data (Additional file 2: Table S21) from the S0, S1, S2 and S3 samples to identify key genes in SR development (Additional file 1: Figure S13a). Comparing to S0, we identified 211, 718, and 791 upregulated genes in S1, S2 and S3 respectively (Additional file 1: Figure S13c). Notably, the starch and sucrose metabolism KEGG pathway ranked respectively the top enrichment for the three upregulated gene sets (Additional file 2: Table S22). We found that several key genes involved in starch metabolism, including *beta-amylase*, *AGPase* (ADP glucose pyrophosphorylase), *SSS* (soluble starch synthase), *SBE* (starch branching enzyme) and *GBSS* (granule bound starch synthase), were upregulated in one or two groups among S1, S2 and S3 compared with S0 (Additional file 1: Figure S14). And more, there are 109 common upregulated genes during SR development (Additional file 1: Figure S13c). Among these genes, eight genes that belong to starch metabolism pathways [52, 53], including *GPT* (Glucose-6-phosphate translocator) [53–55], *PGM* (Phosphoglucomutase), *ISA* (Isoamylase), *SP* (starch phosphorylase), *DPEP* (4-alpha-glucanotransferase) and 3 *Beta-amylase* genes (Additional file 1: Figure S14). *KNOX* and *MIKC-like* (type II *MADS-box*) genes [45, 46, 56] were also identified among the 109 genes. Notably, among these 109 genes, we also found that two specific protein sporamin were highly expressed (RPKM = 32,145~113,739) (Additional file 1: Figure S15, Additional file 2: Table S23). Sporamin is the major storage protein and accounts for 60 to 80% of the total soluble protein in the sweetpotato SR, and its expression has been shown to be primarily associated with SR [57, 58]. The sporamin highly expressed in *I. trifida* SR which indicated that the sweetpotato sporamin may inherit from *I. trifida.* Besides, KEGG enrichment of the differentially downregulated genes displayed that phenylalanine metabolism and phenylpropanoid biosynthesis were the top two pathways enriched in S1, S2 and S3 compared with S0 (Additional file 1: Figure S13b); and we found that the key genes in lignin biosynthesis, *C4H* (coumarate 4-hydroxylase) and *F5H* (ferulate − 5 -hydroxylase) [59], were downregulated from ARs swelling to ISRs, and kept lowly expression in the process of SRs enlargement (Additional file 1: Figure S16, Additional file 2: Table S24). These results indicated that starch accumulation is negatively correlated with lignin formation, and it may be regulated by some metabolism pathways.

To further investigate the key genes responding to SR starch accumulation, we used the starch contents of the dried roots of 200 true F1 individuals and two parents for QTL analysis with a GBS genetic map (Additional file 1: Figures S17 and S18). We obtained five QTLs, which were all located in chromosome 3, including 39 genes (Additional file 2: Table S25). Four of them were upregulated, and one was *BMY11* (Itr.Sc0000035.86, with identity 47.12% to *beta-amylase 1*, named *BMY11*) (Fig. 3b). Notably, among the four upregulated genes, *beta-amylase* gene was identified above among both the expanded gene families in the genome and the differentially upregulated genes. We examined all the members of this gene family in *I. trifida* and four other plants, i.e. *I. nil*, *S. lycopersicum*, *C. canephora*, and *A. thaliana*, and compared them. We found that the *beta-amylase* gene number in *I. trifida* was higher than that in non-SR-forming plants (Additional file 1: Figure S19). In the *beta-amylase* phylogenetic tree, *I. trifida* possessed the same or more number of *beta-amylase* gene members than *I. nil* expect two clade, which were the clade of *BMY11*, containing two *I. nil* genes (INIL06g37601 and INIL06g37600) due to its specific tandem duplication (the distance of these two genes are 3350 bases), and the clade containing three *I. nil* genes (INIL15g27806, INIL15g27808 and INIL10g41052) (Fig. 3c).

### *BMY11* and SR development

**Fig. 4:**
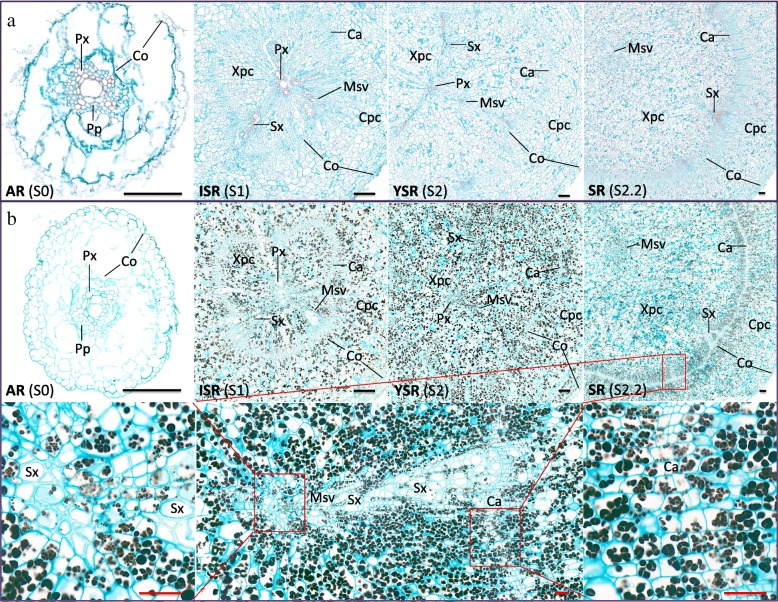
Anatomic structures at different stages of SR development. **a** The anatomic structures of SR at different developmental stages. Safranin-Fast Green staining shows the cutinized cell walls in red and the cellulosic cell walls in green. The diameters of the AR, ISR, YSR and SR (S2.2) were 0.7 mm, 2.6 mm, 5.4 mm and 11.2 mm, respectively. Px, protoxylem; Co, cortex; Pp, primary phloem; Xpc, xylem parenchyma cell; Sx, secondary xylem; Msv, meristem surrounding vessels; Ca, cambium; Cpc, cortex parenchyma cell. **b** The images above show iodine-potassium iodide staining of starch granules in different stages of SR growth. Blue-black and brownish-black dots represent the starch granules. The images below show partially enlarged micrographs: the middle shows an image of the tissue near the cambium and meristem surrounding vessels in the SR, the left side shows an enlarged image of the meristem surrounding vessels in the centre, and the right side shows an enlarged image of the cambium. Black bars = 200 μm, red bars = 50 μm

The function of beta-amylase is to break down starch for grain germination, seedling growth, endosperm development and response to abiotic stresses [60], so it is surprising that *beta-amylase* genes, including *BMY11*, were upregulated during the process of Y22 SR development (Fig. 3b, c) although starch eventually accumulated in SR (Fig. 3a). We speculated that *BMY11* may play a special role in SR development. To investigate this role, we sampled Y22 SR, sliced them transversely and further divided them into five sections. The qPCR results of these five sections showed that *BMY11* was expressed throughout the transverse SR sections (Additional file 1: Figure S20). Beta-amylase was reported that it was located in the parenchyma cells and was accompanied by starch granules [60, 61]. To further investigate the effect of *BMY11* on starch synthesis, we used the roots of S0, S1, S2 and S2.2 (stage 2.2, between S2 and S3, and closer to S2) for starch staining. We found that the starch granules in the new cells near the cambium and the meristem surrounding vessels (MSV) are smaller and much more numerous than those in the cells far from these two tissues (Fig. 4). Combined with the above results, we deduced that *BMY11* may split the smaller starch granules in cells, and then starch synthases may recycle them immediately to synthesize larger starch granules. Moreover, this division may also promote starch translocation between cells in the form of degradation products, again, recycling these degradation products to form larger starch granules. These degradation and recycling processes occur at the same time; therefore, the starch content is gradually increased during the process of SR development and gradually contributes to SR swelling (Fig. 3a). However, the expression of *BMY11* in PR and FR were higher than that of in SR, while the starch accumulation of PR and FR were much lower than that of in SR, which indicated that the function of *BMY11* was primarily to degrade the starch and cannot be highly expression in SR; if not, the excessive degradation would be decrease the starch accumulation and lead the root forming to PR or FR (Additional file 1: Figure S20).

We found one homologous transcript (identity = 98.85%) was also identified using microarray hybridization during the process of SR development in sweetpotato var. Guangshu87 (Additional file 2: Table S26) [6]. one *BMY11* (gene ID: TU52177, identity = 99.39%) in the haplotype-resolved sweetpotato assembly [11]. Through full-length transcript identification sweetpotato var. Xushu18, we found that *BMY11* could exist in more than one copy in the cultivated sweetpotato genome (Additional file 2: Table S27). Similar to Y22, the expressions of *BMY11* in PR and FR were higher than that of in SR, which also indicated that it keep an appropriate activity is beneficial to SR swelling. Based on qPCR results, the expression of *BMY11* in the transverse SR sections of Xushu18 was much lower than that in Y22 SR (Additional file 1: Figure S20). These expression differences indicated that the lower expression of *BMY11* may be beneficial for greater starch accumulation and contribute to rapid SR swelling and larger tuberous root formation in cultivated sweetpotato. A similar expression pattern was found in potato, i.e., *beta-amylase* activity in doubled monoploid (derived from a primitive South American cultivar) tubers was 5 to 10-fold higher than that in a diploid breeding line (more closely resembling commercially cultivated tetraploid potato) tubers [3]. Similar phenomena may have occurred due to natural evolution in the beta-amylase gene family and the relative lower expression of this gene might be essential for the tuberous root crops.

## Discussion

In this research, we used a combined strategy to overcome the problem of high heterozygosity and provide a chromosome-scale reference genome sequence of much higher quality than the previous survey results [24] and the recent released genome [28], which could be considered as a better reference for diploid *I. trifida*. The assembled genome enables us to characterize genomic features in this species and compare the genome with other published plant genomes, such as cultivated sweetpotato and its closely related species, *I. nil* [11, 37]. For example, *I. trifida* harbours fewer repeat sequences than *I. nil* and shows more efficient elimination of LTR retrotransposons, resulting in a smaller genome. Using new evidence, we clarify that both *I. trifida* and *I. nil* underwent a WGT instead of the reported WGD [37] long before their divergence. Furthermore, naturally occurring horizontal gene transfer (HGT) in plants has been reported infrequently, but diploid *I. trifida* (including Y22, Contig_2131, identity = 92.66%) commonly contains *Ib*T-DNA2 [62]. This result indicates that diploid *I. trifida*, as a naturally transgenic plant, could have existed at least nearly 1.3 MYA [11]. Sweetpotato also contains *Ib*T-DNA2 [62, 63]; therefore, this sequence may have been inherited from diploid *I. trifida*. All these findings enable a better understanding of the genome evolution of *I. trifida*, and the high-quality genome of Y22 should be considered a valuable resource for investigation of the genome evolution of sweetpotato and of the genus *Ipomoea* in general.

Within the genus *Ipomoea*, sweetpotato is one of the most important crops, with a global annual production of more than 100 million tons. The released haplotype-resolved genome of sweetpotato was generated by anchoring 75.7% of the scaffolds to 15 pseudochromosomes based on gene and sequence synteny between sweetpotato and *I. nil* [11]. This compressed monoploid (~ 836 Mb) was also considered as valuable resources for investigating the complexity of chromosome sequence composition in sweetpotato [39] and this reference sequence for sweetpotato can be improved with long-read sequencing technology, Hi-C sequencing combining with novel assembly algorithm [11, 39, 64]. The hexaploid genome has proven to be very difficult to assemble and impedes its genetic research in genomic level; to find a proper genetic research model could be a short way. As the closest wild relative and putative progenitor, diploid *I. trifida* has been considered a model species for sweetpotato, including genetic, cytological, and physiological analyses [30]. With the high-quality genome of *I. trifida*, we further demonstrated this concept and identified the key genes associated with an important agronomic trait (SR) with extensive evidence: (1) Y22 can be transplanted like sweetpotato and develop SR (Fig. 3a, Additional file 1: Figure S1), and the SRs of both species contained the highly specific protein sporamin expressed [57] (Additional file 1: Figure S15, Additional file 2: Table S23); (2) the comparative transcriptome analysis of four typical roots provided strong evidence to show that upregulation of genes involved in carbohydrate metabolism and downregulation of those involved in stele lignification, an expression pattern similar to that of sweetpotato, played key roles in the development of Y22 SRs [4, 59, 65] (Additional file 1: Figures S14 and S16; Additional file 2: Table S24); (3) the key gene *BMY11* was associated with SR swelling by comparative transcriptomics and QTL mapping, and *BMY11* has a similar expression pattern in sweetpotato (Additional file 1: Figure S20) [61]. All these findings in our work could accelerate starch biosynthesis study in the genus *Ipomoea*, and the diploid *I. trifida* var. Y22 could be considered an ideal model for future studies of sweetpotato SR development.

## Conclusions

We generated the chromosome-scale genome sequence of SR-forming diploid *I. trifida* var. Y22 with high heterozygosity (2.20%) by integrating whole-genome shotgun reads, single-molecule long reads (PacBio RS II) and GBS genetic maps; and this genome provides a better resolution to its genomics feature. Comparative genomics analysis showed that a whole-genome triplication event occurred in diploid *Ipomoea* genome, and enables a better understanding the genome evolutions of this species. We found that the key gene *BMY11* (with identity 47.12% to *beta-amylase 1*) may contribute to starch accumulation and SR development by combining with the analysis of gene family expansion, QTL mapping, differentially gene expression profiling, morphology and structure of SRs and qPCR of *BMY11*. Sweetpotato SR development genes can be identified in *I. trifida* and these genes perform similar functions and patterns, showed that the diploid *I. trifida* var. Y22 could be considered an ideal model for the studies of sweetpotato SR development.

## Methods

### Plant materials and sequencing

Y22 (Additional file 1: Figure S1a) is a clone of diploid *I. trifida* seeds (CIP No.: PC98_1 (698014), female parent 2X P96124.5, male parent PC) with good SR-forming characteristics. Y25 (Additional file 1: Figure S1b) is a clone of diploid *I. trifida* seeds (CIP No.: 696153, female parent 2X 6.1, male parent OP) that does not form SR (Additional file 1: Figure S1). Xushu18 is a well-known cultivated sweetpotato variety in China. The plant materials were planted in the experimental greenhouse of the Institute of Biotechnology and Nuclear Technology, Sichuan Academy of Agricultural Sciences (Sichuan AAS), Chengdu, Sichuan province, China, and insect netting and yellow sticky paper were used for strict pest control. The F1 genetic population of diploid *I. trifida* was constructed with Y25 (female parent) and Y22 (male parent).

Y22 was used for the whole-genome sequencing and assembly. High-quality genomic DNA was isolated from the leaves using the Qiagen DNeasy Plant Mini Kit (Qiagen, Valencia, CA, USA). First, PE libraries, with insert sizes of 230 bp, 350 bp and 500 bp, were constructed using the NEBNext Ultra DNA Library Prep Kit following the manufacturer’s instructions. Then, mate-pair libraries, with insert sizes of 2 Kb, 5 Kb, 10 Kb and 20 Kb, were generated through circularization by Cre-Lox recombination [66]. Synthetic long reads were generated by Moleculo chemistry. All the above libraries were subjected to the Illumina HiSeq 2500 platform to produce PE 2 × 125 bp reads. Second, the RS II platform with PacBio P6-C4 chemistry was used to generate single-molecule long reads.

For starch content testing and transcriptome sequencing, we took cuttings from Y22 and transplanted them in sandy soil at the Southern Experiment Station (SES) of Sichuan AAS, Sanya, Hainan province, China. The four typical stages of SR development were AR, ISR (diameter = ~ 2 mm), YSR (5–8 mm) and MSR (>20 mm). To examine these stages, the roots were sampled at approximately 25 (S0), 50 (S1), 85 (S2) and 120 (S3) days after transplantation (DAT) (Fig. 3a). Each sample consisted of three repeats. Starch content was tested according to Chinese Testing Standard NY/T11–1985. Total RNA was extracted using TRIzol reagent (Invitrogen) and treated with RNase-free DNase I (Promega, USA). The corresponding RNA-seq libraries were generated using the NEBNext Ultra RNA Library Prep Kit (NEB, USA). Then, all the libraries were sequenced by the Illumina HiSeq Xten platform.

For tag-based sequencing, the F_1_ population (Y25 × Y22) was planted at the SES of Sichuan AAS, Sanya, China. Genomic DNA was extracted from fresh leaves of the individual F_1_ plants and parents using a DNA extraction kit (Tiangen Biotech Co. Ltd., Beijing) and dissolved in 1× TE buffer (10 mM Tris-HCl and 1 mM EDTA, pH 8.0). The individuals were randomly selected from the F_1_ population and confirmed by simple sequence repeat (SSR) markers; the SSRs were designed by a Perl script according to the assembled Y22 genome (Additional file 1: Figure S4 and Additional file 2: Table S5). A total of 202 GBS libraries, including two parent libraries and 200 F1 individual libraries, were prepared according the reference method [32]. Finally, PE sequencing was performed on the selected tags using the Illumina HiSeq Xten platform.

For Xushu18 transcriptome sequencing, we sampled the flowers, stems, leaves and SRs, extracted the total RNA using TRIzol reagent (Invitrogen) and treated it with RNase-free DNase I (Promega, USA). The RNA concentration was determined by Nanodrop (Thermo Scientific), and the different RNAs were mixed in equal proportions to construct the RNA-seq library. The corresponding RNA-seq libraries were generated using the NEBNext Ultra RNA Library Prep Kit (NEB, USA). Then, the libraries were sequenced on the Illumina HiSeq 2500 and PacBio RS II platforms.

### Genome assembly and construction of pseudochromosomes

To obtain high-quality reads, we first filtered all the following reads: (a) reads that contained ‘N’ as more than 10% of the nucleotides; (b) reads that contained adapter sequences; (c) duplicated reads generated by PCR amplification. After quality control, all of the PE and mate-pair reads were assembled with the software Platanus [67] with the default parameters. Second, Illumina Moleculo synthetic long reads were used to perform gap filling with the program PBJelly [68] with the default parameters. Third, PacBio single-molecule long reads were used to further extend the sequence continuity. Fourth, HaploMerger [69] was employed to reduce the sequence redundancy caused by heterozygosity.

The assembled Y22 scaffolds were used as a reference genome to identify SNPs (single nucleotide polymorphism) in Y22, Y25 and their progeny. Variant calling was performed for all samples using the GATK [70] software. The parental polymorphic markers were classified into eight segregation patterns (ab × cd, ef × eg, hk × hk, lm × ll, nn × np, aa × bb, ab × cc and cc × ab). For the F_1_ population, segregation patterns were chosen for the genetic map [71]. Prior to map construction, the markers with segregation distortion (*p* < 0.05), integrity (> 75%), or abnormal bases were filtered. The remaining markers were converted to bin markers using an in-house script. Linkage groups were constructed according to physical position with the JoinMap 4.0 software [72] and determined using a minimum LOD value of 5.0 and a maximum recombination of 45%. Because a large number of segregating SNP loci were involved in the present linkage analysis, the Kosambi mapping function was used to translate recombination frequencies into map distances. The final marker order of each linkage group was verified by the software program RECORD [73]. The parents, Y25 and Y22, were sequenced with average depths of 29.11× and 24.68×, respectively, which were higher than those of the progeny (average 11.77×). In total, 489,692 and 178,112 SNP loci were detected in Y25 and Y22, respectively. The final map consisted of 6306 bin markers containing 15,526 SNPs spanning 3156.55 cM in 15 linkage groups (Additional file 1: Figure S5 and Additional file 2: Table S6). The average genetic distance between SNP markers was 0.50 cM. Then, the scaffolds were anchored to pseudochromosomes according to the locations of the markers using the constructed linkage map (Additional file 1: Figure S6).

### Assembly validation

To assess the accuracy of the assembled genome sequences, we selected the small-fragment library reads and used BWA software [74] to map the reads to the assembled genome. Subsequently, we calculated the mapping rate, the coverage degree and the genome depth. To assess gene structure integrity in the assembly, the transcripts were assembled by Trinity [33] with the parameters -ss 0.5 -jc 0, −minkmercov 2 -minglue 2. The EST sequences were aligned to the assembled genome using BLAT [75] with the default parameters. CEGMA (Core Eukaryotic Genes Mapping Approach) [35] was used to define 248 conserved genes that were also used to evaluate the completeness of gene sequences in the final assembly.

### Genome annotation

Repetitive sequence annotation methods were classified into homologous sequence alignment and ab initio prediction. Homologous sequence alignment methods were based on the repeated sequence database Repbase [76]. We used RepeatMasker and RepeatProteinMask [77] to identify sequences that were similar to known repeat sequences. We also used LTR_FINDER [78], Piler [79], RepeatScout [80], and RepeatModeler (http://www.repeatmasker.org/RepeatModeler/) to build the de novo repeat database. Then, we used RepeatMasker [77] to identify repeats according to the established repeat database.

Gene prediction was based on an integration of de novo prediction, homology-based prediction and RNA-seq prediction. Gene structure de novo prediction was carried out using Augustus [81], GlimmerHMM [82], GeneScan [83], Geneid [84] and SNAP [85] software. Homology-based prediction included protein-based homology searches from closely related or model species. In total, we used 11 homologous species, including *Arabidopsis thaliana*, *Solanum tuberosum*, *Solanum lycopersicum*, *Capsicum annuum*, *Sesamum indicum*, *Beta vulgaris*, *Vitis vinifera*, *Manihot esculenta*, *Nelumbo nucifera.*, *Raphanus sativus* and *Solanum pennellii.* Other evidence supporting this prediction includes the homologous EST or cDNA data that we used to align the predicted gene structure by BLAT [75].

The RNA-seq prediction used experimental RNA-seq data to predict genes. Based on the above prediction results and combined with the transcriptome comparison data, the gene set predicted by each method was integrated into a non-redundant system using EVidenceModeler (EVM) [86]. We used PASA [86] and the transcriptome assembly results to correct the results of the EVM annotation and to add information such as UTRs and splice variants to obtain the final gene set. Putative gene functions were assigned according to the best match of the alignments using BLASTP (E-value≤1e-5) to four databases: InterPro, KEGG, Swiss-Prot and TrEMBL. We identified candidate ncRNAs in the assembled *I. trifida* genome by comparing them with known ncRNA libraries or by structural prediction.

### Phylogenetic analysis

The protein sequences of seven plant species, including *A. thaliana*, *M. esculenta*, *I. nil*, *C. canephora*, *O. sativa*, *S. lycopersicum* and *S. tuberosum*, were downloaded. Then, the gene set of each species was filtered as follows: (a) when multiple transcripts were present in one gene, only the longest transcript in the coding region was used for further analysis; (b) genes encoding proteins of less than 30 amino acids were filtered out. Then, we evaluated the similarity between the protein sequences of all the species through BLASTP with the E-value 1e-5. The protein datasets of all seven species and the Y22 protein dataset were clustered into paralogous and orthologous datasets using the program OrthoMCL [87] with the inflation parameter 1.5.

After gene family clustering, we aligned all 1930 single-copy gene protein sequences by MUSCLE [88] and combined all the alignment results to create an alignment supermatrix. Then, an eight-species phylogenetic tree was constructed using RAxML [89] with the maximum likelihood method, and the number of bootstrap samples was set to 100. *O. sativa* was designated as an outgroup of the phylogenetic tree. Using the single-copy gene families, the divergence time estimates were made using MCMCtree in the PAML [90, 91] package. The MCMCtree running parameters were as follows: burn-in: 5,000,000, sample-number: 1,000,000, sample-frequency: 50. The time correction points were *O. sativa* and *A. thaliana*, 140–200 MYA; *A. thaliana* and *S. tuberosum*, 101–156 MYA; *C. canephora* and *S. lycopersicum*, 83–89 MYA; *S. tuberosum* and *S. lycopersicum*, 7.2–7.4 MYA. All time correction points were derived from the TimeTree website (http://www.timetree.org/).

### Genome synteny and whole-genome duplication (WGD)

BLASTP was implemented with an E-value of 1e-5 between different species, including *I. trifida* to *I. trifida*, *S. tuberosum* to *S. tuberosum*, *I. nil* to *I. nil*, *C. canephora* to *C. canephora*, *I. trifida* to *S. tuberosum*, *I. trifida* to *I. nil* and *I. nil* to *S. tuberosum*. MCscan [92] was used to search for collinear segments within each comparison pair. Then, the four-fold transversion (4dTv) ratio for each gene pair in the block of each comparison group was calculated from concatenated nucleotide alignments with MUSCLE [88]. WGD was estimated using the 4dTv ratio distribution. The synteny blocks between chromosomes were visualized by Circos.

### Gene expression profiling of roots

After sequencing, raw RNA reads were filtered and trimmed to yield clean reads. All RNA reads were mapped to the Y22 genome by TopHat2 [93] with the following parameters: --max-intron-length 500,000, −-read-gap-length 10, −-read-edit-dist 15, −-max-insertion-length 5 and --max-deletion-length 5. The read count of each sample was calculated by HTSeq [94]. Then, RPKM was calculated by its definition (reads per kilobase per million mapped reads). Differentially expressed genes of different comparison groups were defined using DESeq [95] with ajusted Pvalue(Padj) < 0.05.

### Definition and identification of starch metabolism genes in Y22

We defined and identified starch metabolism genes using a combined method. Starch metabolism genes were defined as upstream or downstream genes involved in starch synthesis or in the starch synthesis pathway. The key enzyme genes in this study were identified by the following method: first, the protein sequences of these genes in *A. thaliana* were downloaded from NCBI (https://www.ncbi.nlm.nih.gov/). The candidate genes were identified by BLASTP with an E-value of 1e-5. Second, the candidate genes were filtered by identity, and overlaps were removed. Protein domains of homologous species and Y22 candidate genes were predicted by PFAM (http://pfam.xfam.org/). Only the candidate genes with the same protein domains as those in homologous species were kept. Transcription factors were identified by iTAK software [96].

### Phylogenetic reconstruction of beta-amylase (BMY) gene family

For the identification of *BMY* gene family members in *I. trifida*, *I.nil* genome and other genomes, the protein sets in these species were aligned against to known beta-amylase proteins from *A. thaliana* by BLASTp. For each protein, only the alignment with an E-value of 1e-5 and identity > 50% was retained. These genes with retained alignments to responding *A. thaliana* proteins were further required to possess the PF01373 PFAM domains and then were considered as initial candidate *BMY* genes in these genomes. In order to eliminate the inaccuracy of this artificial selection condition (identity > 50% might be too strict for *I. trifida* and *I.nil*), we redo the *BMY* gene identification for these species by replacing the known *A. thaliana* beta-amylase proteins with their respective protein sequences of initially detected *BMY* genes and then follow the same process. Then, all of retained *BMY* genes were aligned to each other using MUSCLE and the phylogenetic tree for *BMY* genes was constructed using RAxML with the maximum likelihood method.

### QTL mapping

The whole roots of 200 F1 individuals were dug up and washed at 130 DAT (Additional file 1: Figure S17). The whole roots were cut into 3 mm slices or 3 cm pieces and dried at 80 °C to a stable weight. The whole roots were comminuted until all fragments would pass through a 0.17 mm mesh sieve. Then, all the samples were sent to the Analysis and Determination Centre of Sichuan AAS for starch testing according to Chinese Testing Standard NY/T11–1985. The starch contents of the dry roots ranged from 13.91 to 47.32% (Additional file 1: Figure S18). The root starch content data were constructed into a data matrix, which was used for QTL analysis by the Windows QTL Cartographer V2.5 software [97]. Phenotypic variance and QTL detection were calculated with a multiple QTL mapping model (MQM). The phenotypic threshold of LOD scores for evaluating the statistical significance of QTL effects was determined using 1000 permutations and a threshold of *p* = 0.05. LOD values 3.0 and above were considered to indicate QTL loci.

### qPCR

The total RNA isolated by the RNAprep Pure Plant Kit (Tiangen Biotech, Beijing) was used to synthesize the first-strand cDNA by oligo (dT)18 and random hexamer primers with the ReverTra Ace qPCR RT Master Mix (Toyobo, Japan). Quantitative real-time reverse transcription PCR (qPCR) was carried out using diluted cDNA and SYBR® Green Real-time PCR Master Mix (Toyobo, Japan) in the Bio-Rad iCycler MyiQ Real-Time PCR System. The qPCR cycle profile included one cycle of 95 °C for 30 s, followed by 40 cycles of 95 °C for 5 s, 58 °C for 30 s, and a final melt curve profile (65–95 °C). The changes in gene expression were calculated relative to Actin using the 2^−ΔΔCt^ method (Additional file 2: Table S28). Each data point represents the average of three repeats.

### Root anatomic structure

The fresh roots were fixed in 4% neutral-buffered formalin for 48 h and then dehydrated and embedded in paraffin wax. Eight-micrometre sections were cut and placed on silane-coated slides to fix the samples. After drying at 60 °C, the sections were dewaxed and rehydrated. The sections were prepared for starch staining and Safranin-Fast Green staining. For starch staining, the sections were stained with I-KI. For Safranin-Fast Green staining, the sections were stained with the Safranin solution for 60–120 min and destained with gradient alcohol. The sections were placed into Fast Green solution for 30–60 s, dehydrated and mounted with resin. All the images were collected using a 3DHISTECH scanner (Pannoramic MIDI) and the software CaseViewer was used to view the image data (https://www.3dhistech.com/caseviewer).

## Additional files

Additional file 1: **Figure S1.** Diploid *Ipomoea trifida* and sweetpotato. (a) Whole plant of Y22, showing storage root (SR) formation. Y22 is a clone of diploid *I. trifida* seeds (CIP No: PC98_1 (698014), female parent 2X P96124.5, male parent PC). (b) Whole plant of Y25, which does not form SRs. Y25 is a clone of diploid *I. trifida* seeds (CIP No: 696153, female parent 2X 6.1, male parent OP). (c) Transverse and longitudinal sections of a pencil root (PR) from Y25 and a SR from Y22; the cortex can be easily stripped from the Y22 SR. (d) F1 progeny (clone 3–11) of Y22, which also has strong SR development. (e) The SR of sweetpotato var. Xushu22. Scale bar: 2 cm. **Figure S2.** K-mer analysis for estimating the genome size of *I. trifida*. K = 17. The X-axis shows the depth, and the Y-axis represents the frequency at each depth. **Figure S3.** Heterozygosity assessment using a fitting curve. The light blue curve is consistent with the heterozygosity of the genome. Therefore, the heterozygosity is 2.20%. **Figure S4.** SSR identification of true F1 individuals. M represents the marker. Y25 was the female parent, and Y22 was the male parent. 3–11, 1–6, 2–6, 4–1, 2–3 and 2–7 were the F1 individuals. In the electrophoretic bands, any of the bands with Y22 existed on the basis of the Y25 bands in the progeny is true hybrid. **Figure S5.** The high-density genetic map of *I. trifida*. **Figure S6.** The fifteen pseudochromosomes of *I. trifida*. The scaffolds were anchored to pseudochromosomes according to the locations of markers from the constructed linkage map. The blue pillars represent the chromosomes, which each consist of multiple scaffolds. The green pillars represent the fifteen linkage groups, and the grey lines link the markers from the linkage groups to the physical locations on the chromosomes. **Figure S7.** GC content and mean sequence depth calculated with a 10 k non-overlapping sliding window. The x-axis represents the GC content, and the y-axis represents the average depth with 10-kb non-overlapping sliding windows. The histogram at right represents the average depth distribution, while the histogram above represents the GC content distribution of the *I. trifida* genome. **Figure S8.** GC content and mean sequence depth of the *I. trifida* genome calculated with a 10 k non-overlapping sliding window. The x-axis represents the GC content, and the y-axis represents the density of GC content. **Figure S9.** Gene set evidence supports statistics. The blue circle represents the 29,728 genes predicted de novo, the light blue circle represents the 24,109 genes predicted by RNA evidence, and the red circle represents the 25,618 genes predicted by homology from seven species including *Arabidopsis thaliana*, *Beta vulgaris*, *Capsicum annuum*, *Sesamum indicum*, *Solanum lycopersicum*, *Solanum tuberosum* and *Vitis vinifera*. **Figure S10.** Insertion time distribution of LTR-RTs of *I. trifida* and *I. nil*. The y-axis represents the copy numbers of LTR-RTs and the x-axis represents the insertion time of LTR-RTs. We performed alignment of the sequences between the 5′ and 3′ LTRs using MUSCLE (v3.8.31, http://www.drive5.com/muscle). LTR insertion time (T) was calculated with the formula T = k/2r (divergence between LTRs / substitution per site per year, r = 1.05E-8). **Figure S11.** Collinear blocks between *I. trifida* and *I. nil*. Different colours represent different chromosomes. **Figure S12.** Collinear blocks between *I. trifida* and the haplotype-resolved *I. batatas* genome. Different colours represent different chromosomes. Itr: *I. trifida*, Ib: *I. batatas.* **Figure S13.** Venn diagrams of differentially expressed gene numbers when comparing groups S1 vs S0, S2 vs S0 and S3 vs S0. (a) Venn diagram of all differentially expressed genes, including up- and downregulated genes. (b) Venn diagram of differentially downregulated genes. (c) Venn diagram of differentially upregulated genes in the three comparison groups. The differentially upregulated genes were defined using DESeq with Padj< 0.05. The number 109 indicates the differentially upregulated genes common to all groups. **Figure S14.** SR development and responsive gene regulation in *I. trifida*. (a) A model of the starch synthesis pathway showing the 109 commonly upregulated genes. The small Venn diagram beside each gene represents the differently upregulated gene numbers in the three comparison groups. *GPT*, Glucose-6-phosphate translocator; *PGM*, Phosphoglucomutase; *SBE*, starch branching enzyme; *ISA*, Isoamylase; *AGPase*, ADP glucose pyrophosphorylase; SSS, soluble starch synthase; *SP*, starch phosphorylase; *GBSS*, granule-bound starch synthase; *SuS*, sucrose synthase; *DPEP*, 4-alpha-glucanotransferase; *β-amylase*, *Beta-amylases.* The comparative transcriptome analysis of four typical roots provided strong evidence to show that upregulation of genes involved in carbohydrate metabolism and downregulation of those involved in stele lignification, an expression pattern similar to that of sweetpotato, played key roles in the development of Y22 SR. (b) Expression heatmap of the starch synthesis pathway genes. **Figure S15.** RPKM values of two sporamins in Y22 SRs. **Figure S16.** Heatmap of lignin synthesis genes*.* Ten genes in the lignin synthesis pathway were identified in *I. trifida*. *PAL*, Phenylalanine ammonia-lyase; *C3H*, 4-Coumarate 3-hydroxylase; *C4H*, Coumarate-4-hydroxylase; *CAD*, Cinnamyl alcohol dehydrogenase; *CCR*, Cinnamoyl-CoA reductase; *CCoAOMT*, Caffeoyl-CoA *O*-methyltransferase; *COMT*, Caffeic acid/5-hydroxyconiferaldehyde O -methyltransferase; *F5H*, Ferulate 5-hydroxylase; *4CL*, 4-coumarate: CoA ligase; *HCT*, *p*-hydroxycinnamoyl-CoA:quinate shikimate *p*-hydroxycinnamoyltransferase. **Figure S17.** Representative F1 individuals. 3–11 had typical SR. 1–6, 2–6 and 4–1 had thickened roots (SR or SR-like; the xylem of some roots was partially lignified). 2–3 had pencil roots (PR). 2–7 had fibrous roots (FR). Scale bar: 2 cm. **Figure S18.** Frequency distribution of starch content in dry roots. 13 ≤ 16 means that the starch content was higher than 13% and less than or equal to 16%; the ≤ symbol is used similarly throughout the x-axis labels. **Figure S19.** *wBeta-amylase* gene numbers in five species. **Figure S20.** Expression of *BMY11* in SR. (a) Transverse section of an SR from *I. trifida* var. Y22. The SR was sliced transversely and further divided into five sections: section 1 was the outer section of the cortex including the epidermis (SC1), section 2 included the inner section of the cortex and outermost portion of the xylem (SC2), section 3 was the outer part of the xylem (SC3), section 4 was the middle part of the xylem (SC4), and section 5 was the inner part of the xylem (SC5). PR and FR were used as controls. (b) Transverse section of an SR from sweetpotato var. Xushu18. The SR was sectioned as in (a). Bar = 10 mm. **(c)** qRT-PCR results of *BMY11* in the transverse SR sections of Y22 and Xushu18. (DOCX 5745 kb)

Additional file 2: **Table S1.** Survey statistic results. **Table S2.** Sequencing data statistics. **Table S3.** Statistics of assembly results with only Illumina sequencing data. **Table S4.** Statistics of assembly results after extension with PacBio RS II data. **Table S5.** SSR primer used for identification of true F1 hybrids. **Table S6.** Statistical information of genetic linkage groups. **Table S7.** Coverage statistics of the *I. trifida* genome. **Table S8.** EST sequence evaluation results. **Table S9.** CEGMA evaluation results. **Table S10.** RNA-seq data used for annotation. **Table S11.** Statistical results of gene functional annotations. **Table S12.** Statistical results of non-coding RNAs. **Table S13.** Statistical results of repeated classification. **Table S14.** Summary of the plant species and assemblies/gene models used in this study. **Table S15.** Comparison of repeat contents between *I. trifida* and *I. nil*. **Table S16.** Statistics of LTR numbers. **Table S17.** Lengths of syntenic blocks and block repeat sequences. **Table S18.** Chromosome duplication test results for Y22. **Table S19.** KEGG enrichment results for the genes expanded in *I. trifida*. **Table S20.** KEGG enrichment results for the genes contracted in *I. trifida*. **Table S21.** Sequencing data statistics of RNA from S0 to S3. **Table S22.** KEGG pathways of upregulated genes. **Table S23.** Blast results of the specific protein sporamin in the *I. trifida* assembly. **Table S24.** KEGG pathways of downregulated genes. **Table S25.** QTL mapping results. **Table S26.** Results of BMY11 microarray hybridization during the process of SR development in sweetpotato var. Guangshu87. **Table S27.** Blast results of *BMY11* in the full-length transcripts of sweetpotato var. Xushu18. **Table S28.** qPCR primers used to amplify *BMY11*. (DOCX 75 kb)
