# Supplementary material for: The wild sweetpotato (Ipomoea trifida) genome provides insights into storage root development
Source: BMC Plant Biol. 2019 Apr 1;19:119. doi: 10.1186/s12870-019-1708-z (PMC6444543; doi:10.1186/s12870-019-1708-z)
Supplement: Supplementary file 1 — Figure S1. Diploid Ipomoea trifida and sweetpotato. (a) Whole plant of Y22, showing storage root (SR) formation. Y22 is a clone of diploid I. trifida seeds (CIP No: PC98_1 (698014), female parent 2X P96124.5, male parent PC). (b) Whole plant of Y25, which does not form SRs. Y25 is a clone of diploid I. trifida seeds (CIP No: 696153, female parent 2X 6.1, male parent OP). (c) Transverse and longitudinal sections of a pencil root (PR) from Y25 and a SR from Y22; the cortex can be easily stripped from the Y22 SR. (d) F1 progeny (clone 3–11) of Y22, which also has strong SR development. (e) The SR of sweetpotato var. Xushu22. Scale bar: 2 cm. Figure S2. K-mer analysis for estimating the genome size of I. trifida. K = 17. The X-axis shows the depth, and the Y-axis represents the frequency at each depth. Figure S3. Heterozygosity assessment using a fitting curve. The light blue curve is consistent with the heterozygosity of the genome. Therefore, the heterozygosity is 2.20%. Figure S4. SSR identification of true F1 individuals. M represents the marker. Y25 was the female parent, and Y22 was the male parent. 3–11, 1–6, 2–6, 4–1, 2–3 and 2–7 were the F1 individuals. In the electrophoretic bands, any of the bands with Y22 existed on the basis of the Y25 bands in the progeny is true hybrid. Figure S5. The high-density genetic map of I. trifida. Figure S6. The fifteen pseudochromosomes of I. trifida. The scaffolds were anchored to pseudochromosomes according to the locations of markers from the constructed linkage map. The blue pillars represent the chromosomes, which each consist of multiple scaffolds. The green pillars represent the fifteen linkage groups, and the grey lines link the markers from the linkage groups to the physical locations on the chromosomes. Figure S7. GC content and mean sequence depth calculated with a 10 k non-overlapping sliding window. The x-axis represents the GC content, and the y-axis represents the average depth with 10-kb non-overlapping sliding [file 12870_2019_1708_MOESM1_ESM.docx]

**Additional file 1**

**
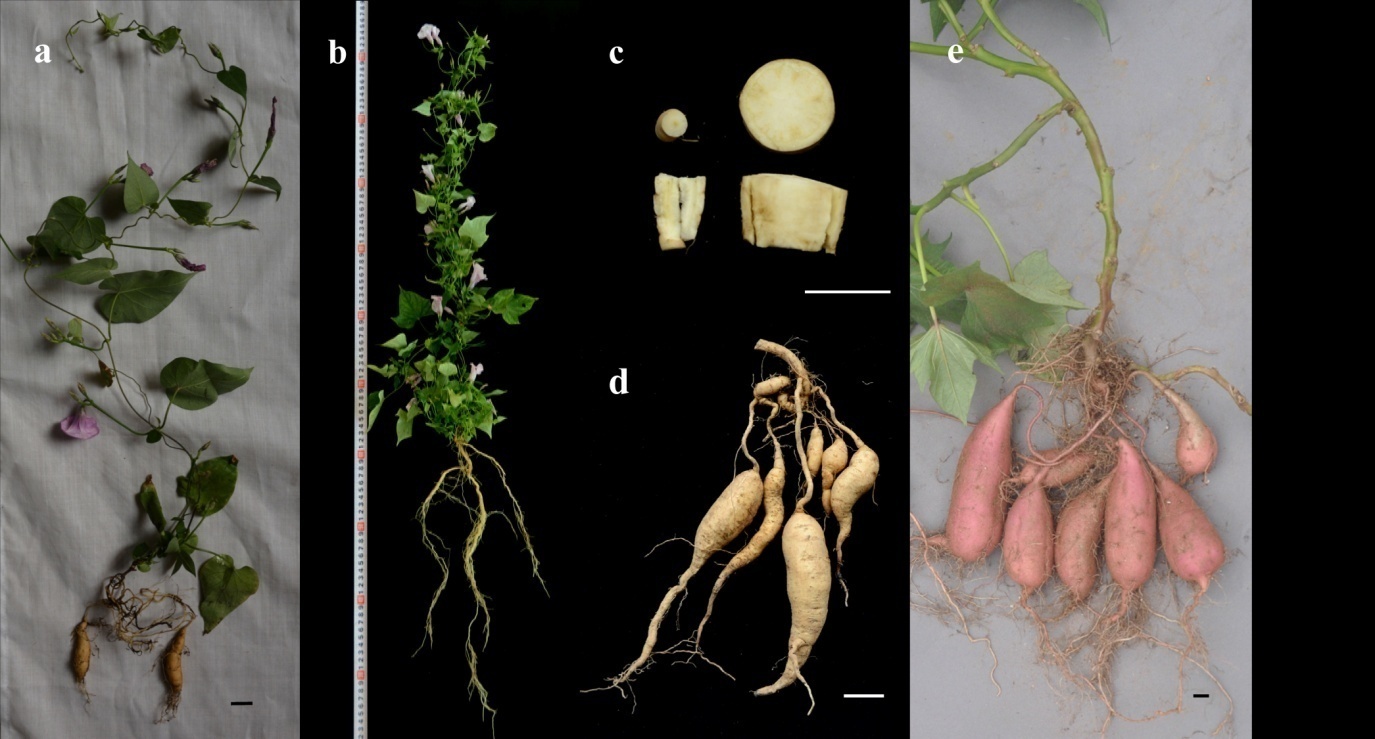
**

**Figure S1. D****iploid *Ipomoea trifida* and sweetpotato.**

**(a)** Whole plant of Y22, showing storage root (SR) formation. Y22 is a clone of diploid *I. trifida* seeds (CIP No: PC98_1 (698014), female parent 2X P96124.5, male parent PC). **(b)** Whole plant of Y25, which does not form SRs. Y25 is a clone of diploid *I. trifida* seeds (CIP No: 696153, female parent 2X 6.1, male parent OP). **(c)** Transverse and longitudinal sections of a pencil root (PR) from Y25 and a SR from Y22; the cortex can be easily stripped from the Y22 SR. **(d)** F1 progeny (clone 3-11) of Y22, which also has strong SR development. **(e)** The SR of sweetpotato var. Xushu22. Scale bar: 2 cm.

**
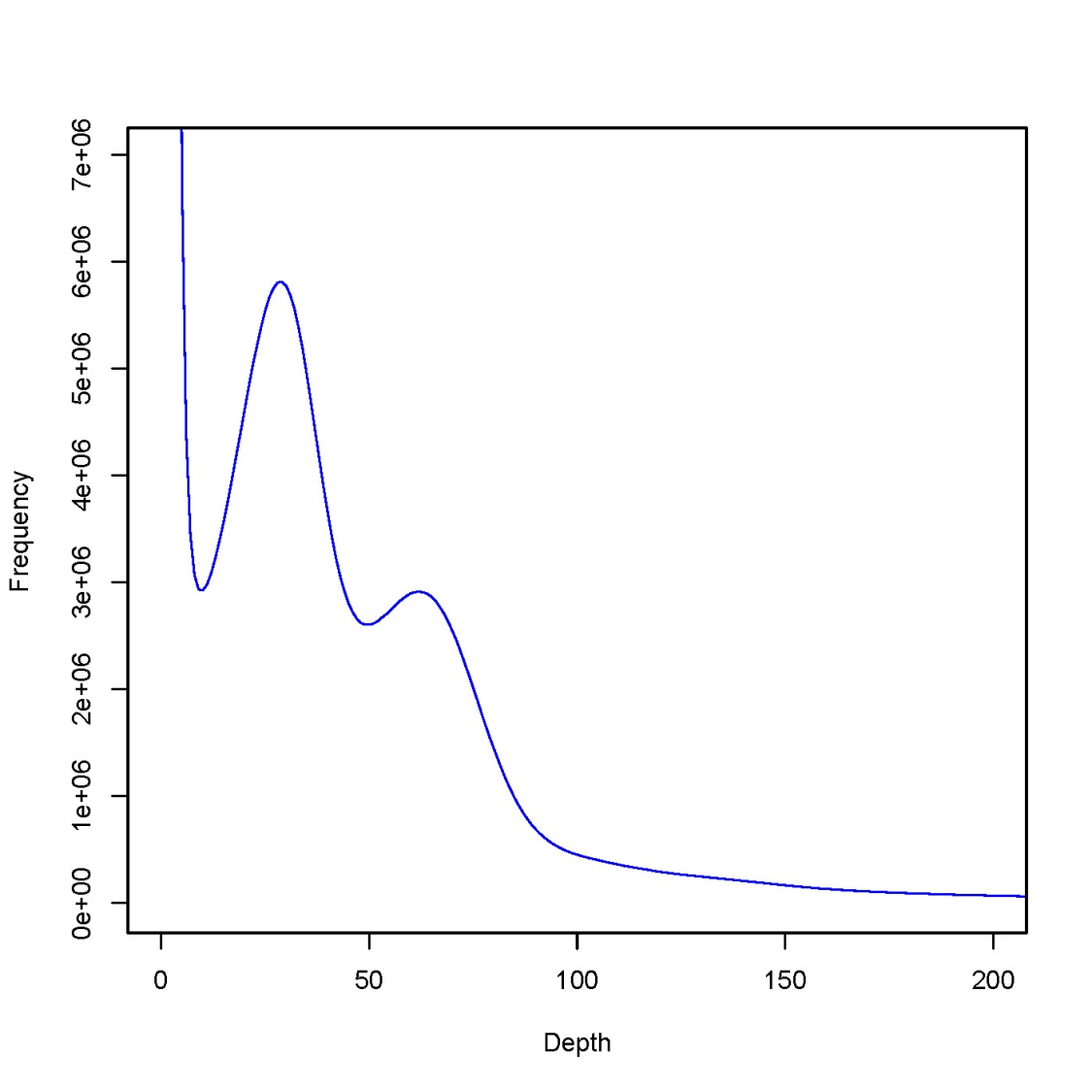
**

**Figure S2****. K-mer analysis for estimating the genome size of** ***I. trifida*.**

K=17. The X-axis shows the depth, and the Y-axis represents the frequency at each depth.


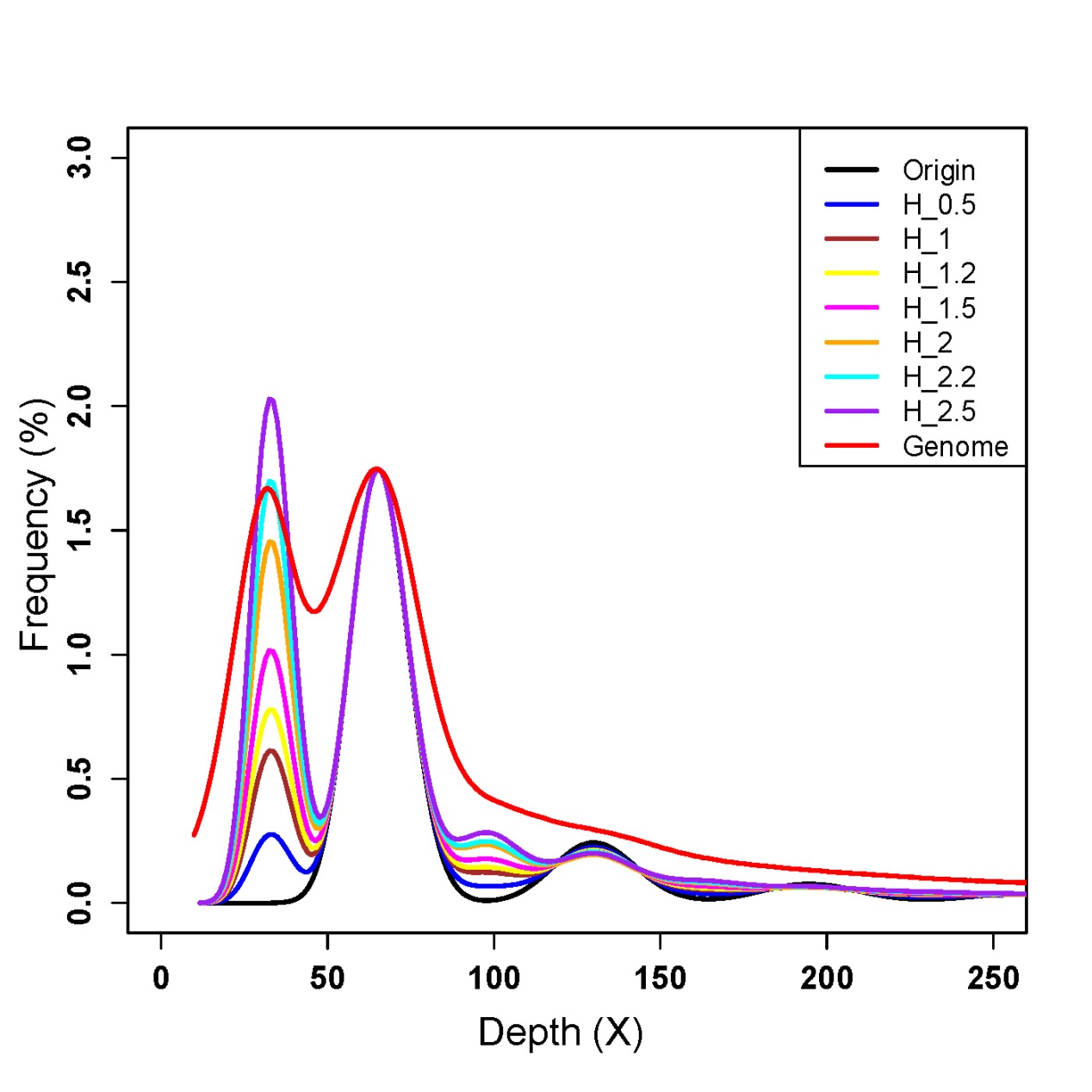


**Figure S3. Heterozygosity assessment using a fitting curve.**

The light blue curve is consistent with the heterozygosity of the genome. Therefore, the heterozygosity is 2.20%.


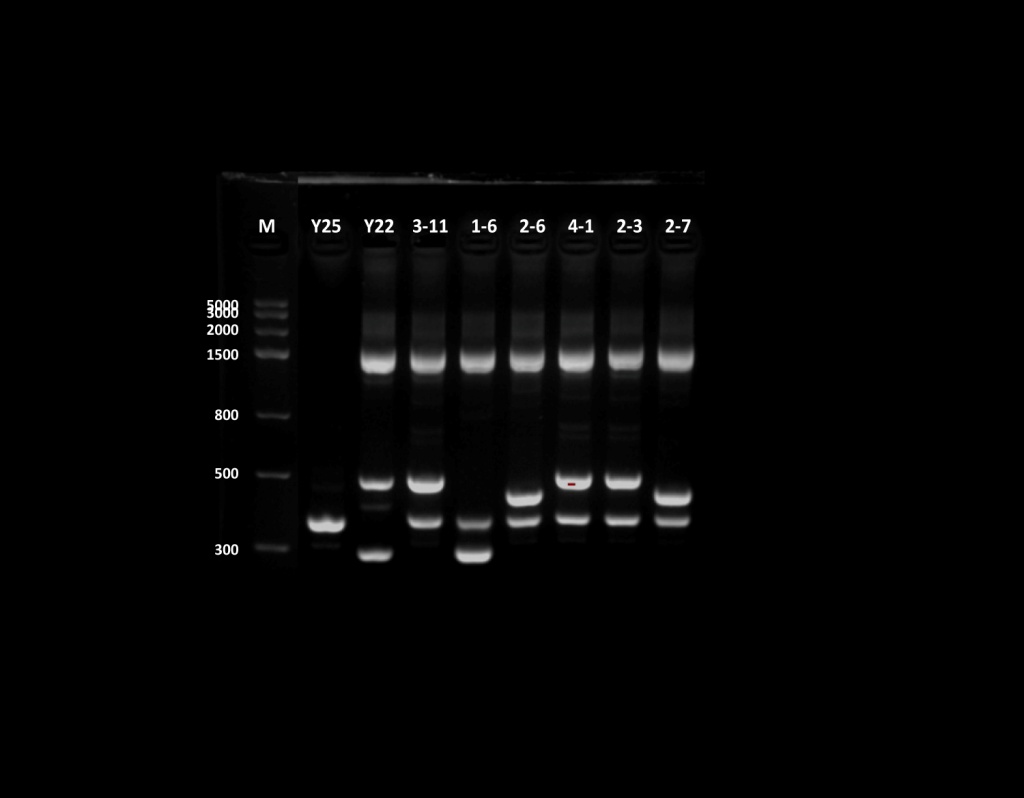


**Figure S4. SSR identification of true F1 individuals.**

M represents the marker. Y25 was the female parent, and Y22 was the male parent. 3-11, 1-6, 2-6, 4-1, 2-3 and 2-7 were the F1 individuals. In the electrophoretic bands, any of the bands with Y22 existed on the basis of the Y25 bands in the progeny is true hybrid.


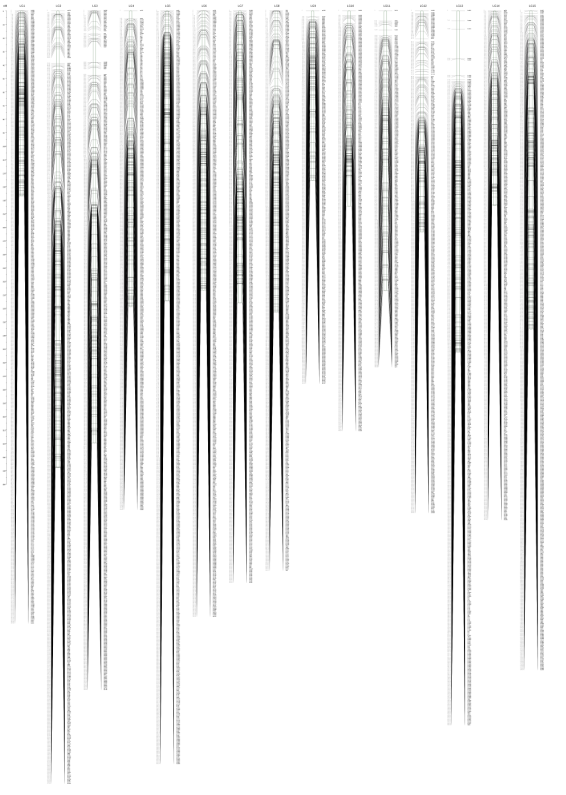


**Figure S5. The high-density genetic map of *I. trifida*.**


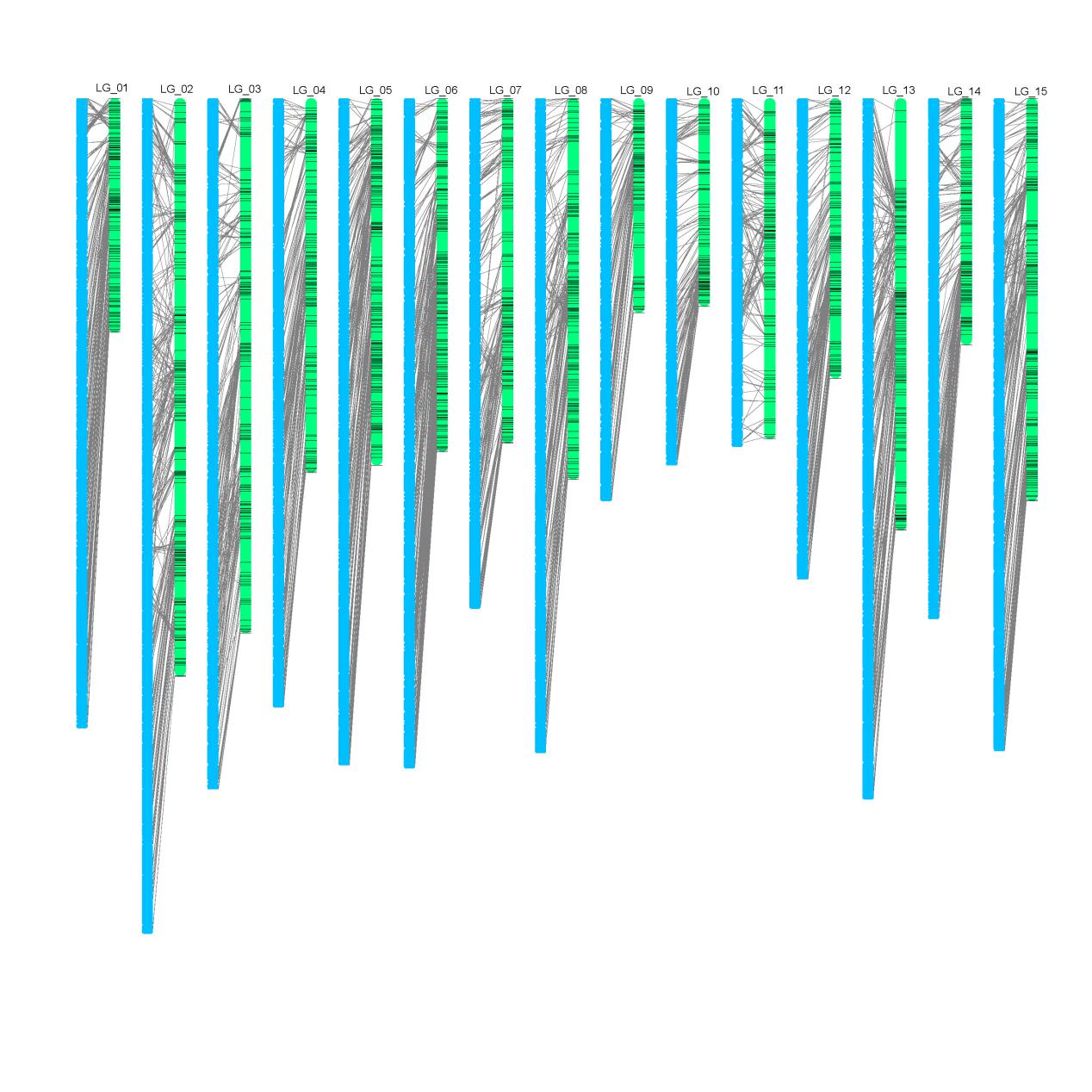


**Figure S6. The fifteen pseudochromosomes of *I. trifida*.**

The scaffolds were anchored to pseudochromosomes according to the locations of markers from the constructed linkage map. The blue pillars represent the chromosomes, which each consist of multiple scaffolds. The green pillars represent the fifteen linkage groups, and the grey lines link the markers from the linkage groups to the physical locations on the chromosomes.


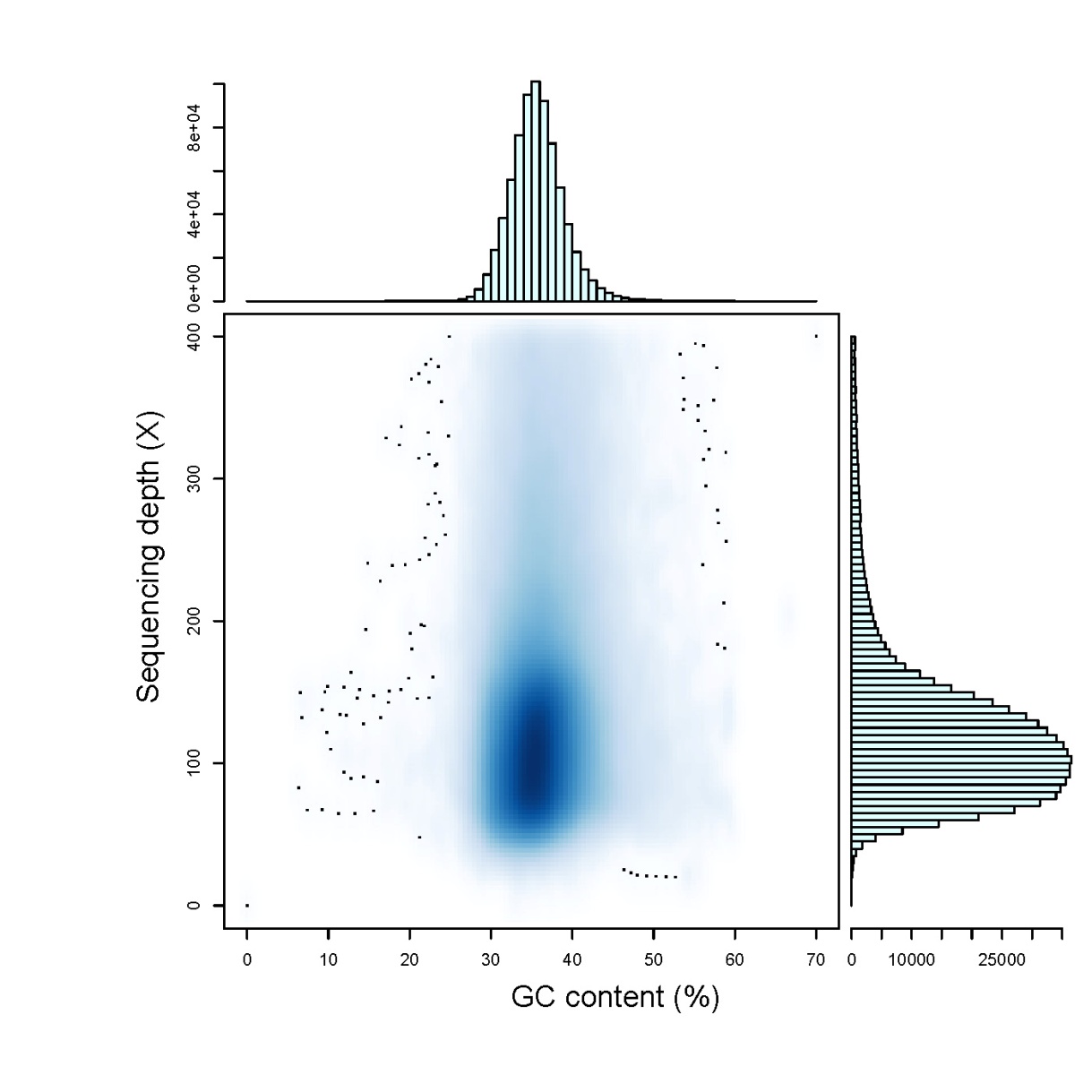


**Figure S7. GC content and mean sequence depth calculated with a 10k non-overlapping sliding window.**

The x-axis represents the GC content, and the y-axis represents the average depth with 10-kb non-overlapping sliding windows. The histogram at right represents the average depth distribution, while the histogram above represents the GC content distribution of the *I. trifida* genome.


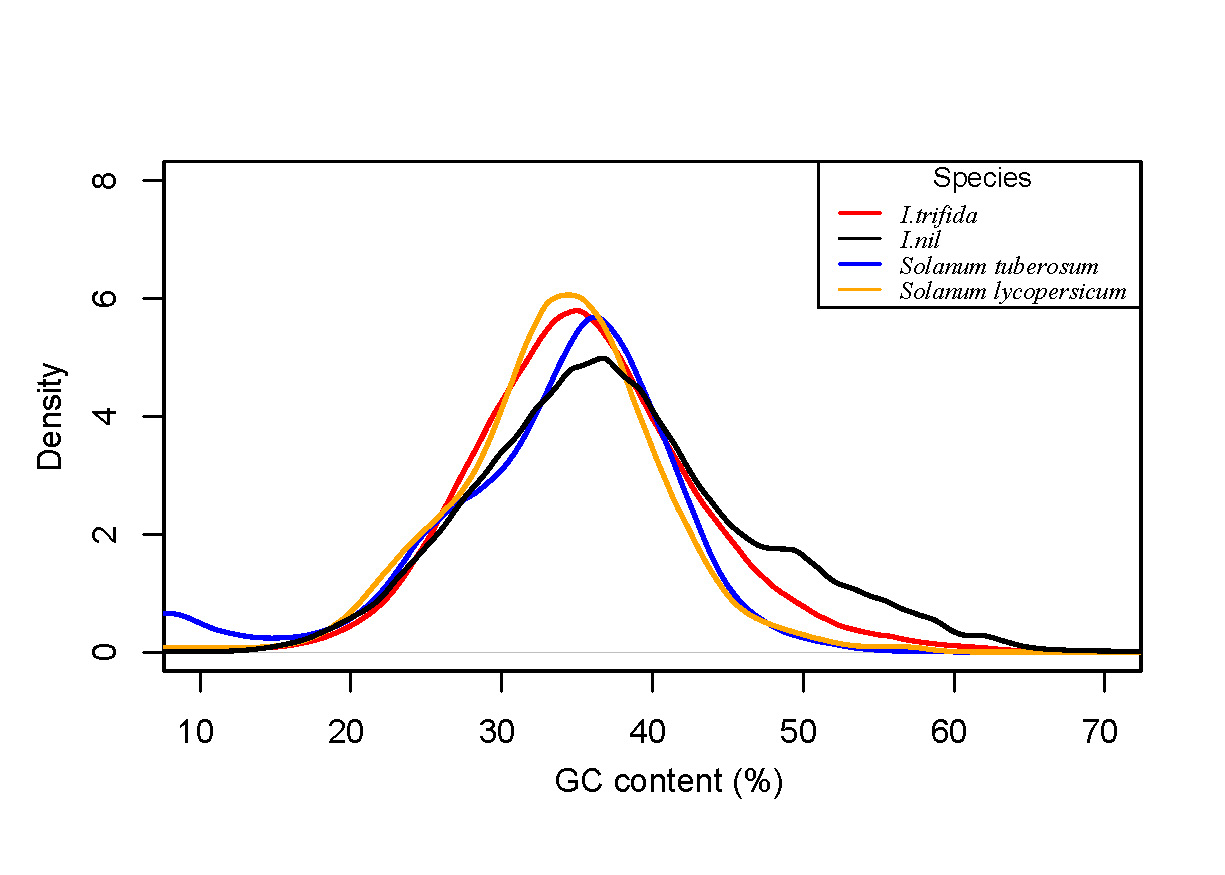


**Figure S8. GC content and mean sequence depth of the *I. trifida* genome calculated with a 10k non-overlapping sliding window.**

The x-axis represents the GC content, and the y-axis represents the density of GC content.

**
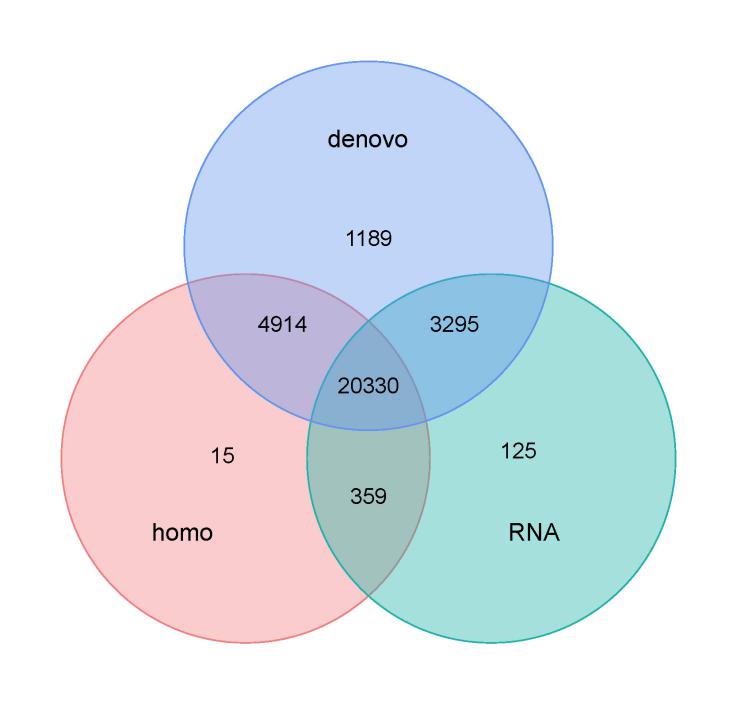
**

**Figure S9. Gene set evidence supports statistics.**

The blue circle represents the 29,728 genes predicted *de novo*, the light blue circle represents the 24,109 genes predicted by RNA evidence, and the red circle represents the 25,618 genes predicted by homology from seven species including *Arabidopsis thaliana*, *Beta vulgaris*, *Capsicum annuum*, *Sesamum indicum*, *Solanum lycopersicum*, *Solanum tuberosum* and *Vitis vinifera*.


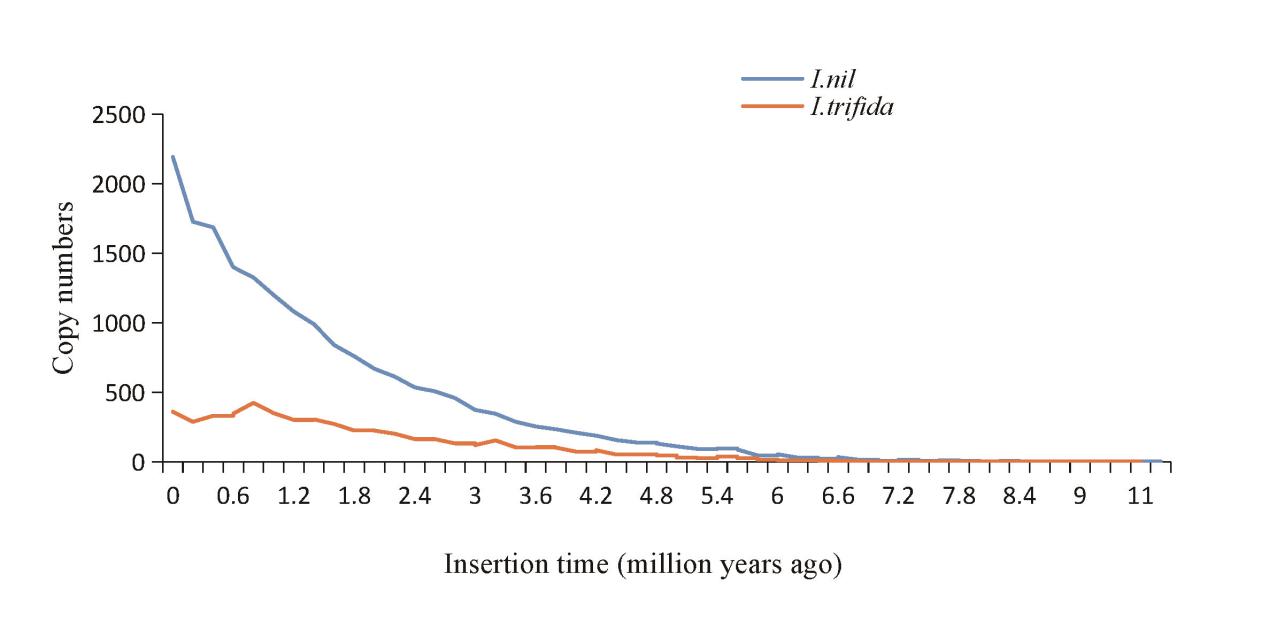


**Figure S10. Insertion time distribution of LTR-RTs of *I.trifida* and *I.nil*.**

The y-axis represents the copy numbers of LTR-RTs and the x-axis represents the insertion time of LTR-RTs. We performed alignment of the sequences between the 5’ and 3’ LTRs using MUSCLE (v3.8.31, http://www.drive5.com/muscle). LTR insertion time (T) was calculated with the formula T=k/2r (divergence between LTRs / substitution per site per year, r =1.05E-8).

**

**

**Figure S11. Collinear blocks between *I. trifida* and *I. nil*.**

Different colours represent different chromosomes.


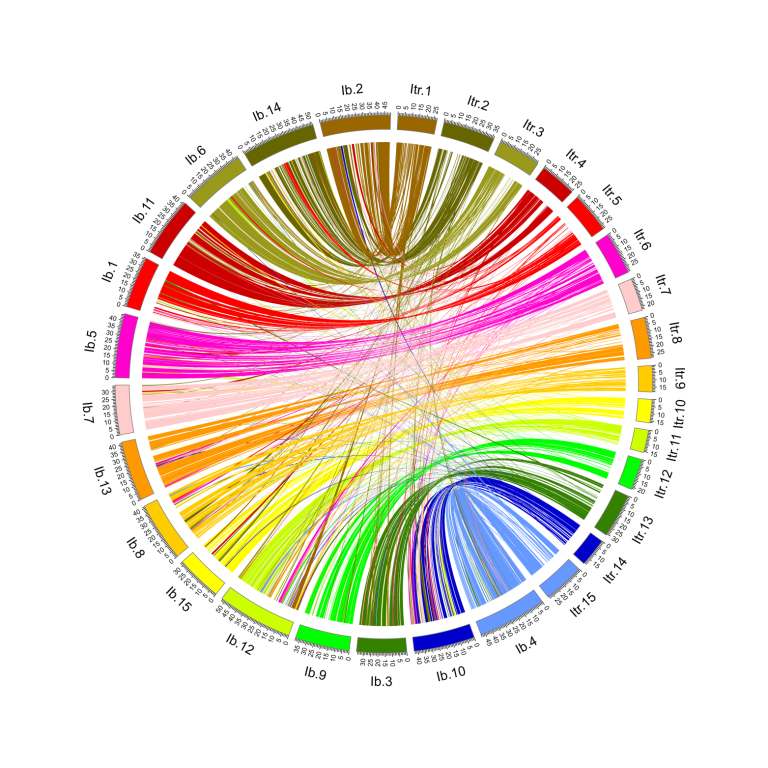


**Figure S12.** **Collinear blocks between *I. trifida* and the haplotype-resolved *I. batatas* genome.**

Different colours represent different chromosomes. Itr: *I. trifida*, Ib: *I. batatas.*


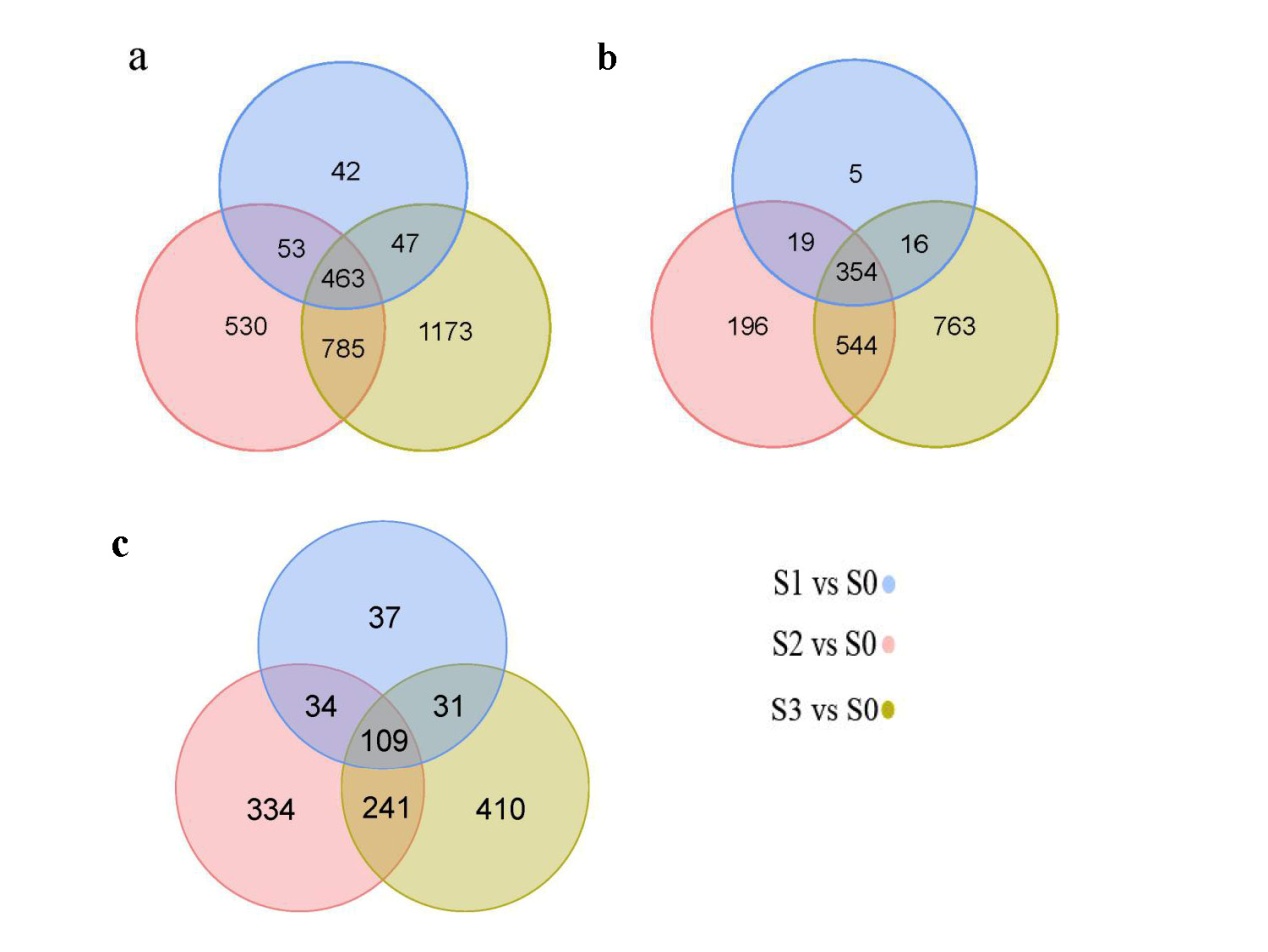


**Figure S13. Venn diagrams of differentially expressed gene numbers when comparing groups S1 vs S0, S2 vs S0 and S3 vs S0.**

**(a)** Venn diagram of all differentially expressed genes, including up- and downregulated genes. **(b)** Venn diagram of differentially downregulated genes. **(c)** Venn diagram of differentially upregulated genes in the three comparison groups. The differentially upregulated genes were defined using DESeq with Padj<0.05. The number 109 indicates the differentially upregulated genes common to all groups.

**Figure S14. SR development and responsive gene regulation in *I. trifida*.**

**(a)** A model of the starch synthesis pathway showing the 109 commonly upregulated genes. The small Venn diagram beside each gene represents the differently upregulated gene numbers in the three comparison groups. *GPT*, Glucose-6-phosphate translocator; *PGM*, Phosphoglucomutase; *SBE*, starch branching enzyme; *ISA*, Isoamylase; *AGPase*, ADP glucose pyrophosphorylase; SSS, soluble starch synthase; *SP*, starch phosphorylase; *GBSS*, granule-bound starch synthase; *SuS*, sucrose synthase; *DPEP*, 4-alpha-glucanotransferase; *β-amylase*, *Beta-amylases.* The comparative transcriptome analysis of four typical roots provided strong evidence to show that upregulation of genes involved in carbohydrate metabolism and downregulation of those involved in stele lignification, an expression pattern similar to that of sweetpotato, played key roles in the development of Y22 SR. (b) Expression heatmap of the starch synthesis pathway genes.

**Figure S15.** FPKM values of two sporamins in Y22 SRs.


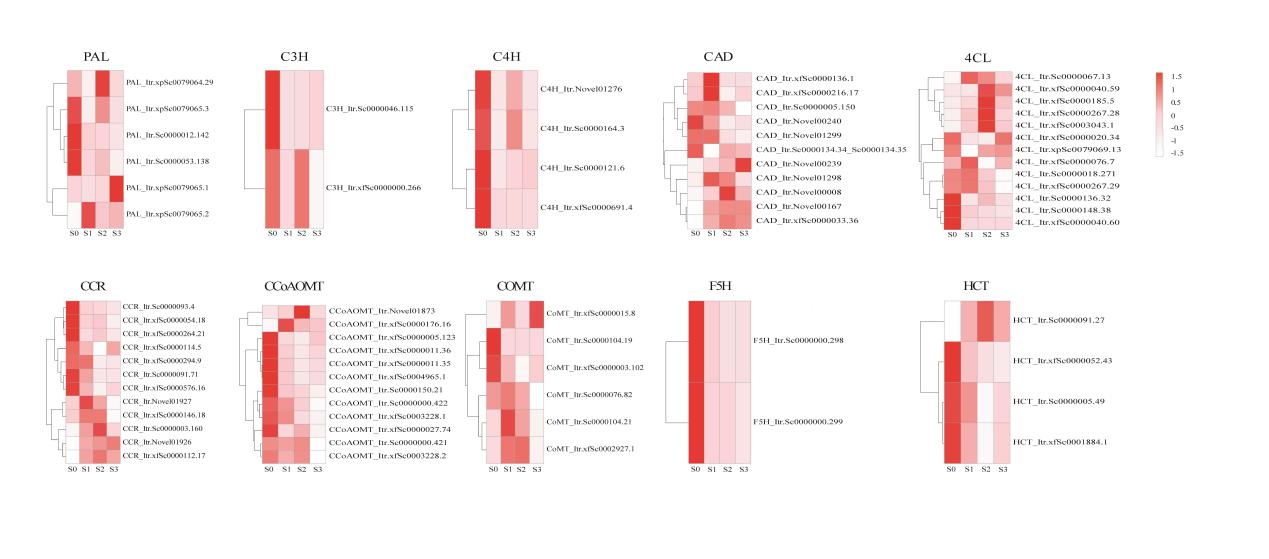


**Figure S16. Heatmap of lignin synthesis genes*.***

Ten genes in the lignin synthesis pathway were identified in *I. trifida*. *PAL*, Phenylalanine ammonia-lyase; *C3H*, 4-Coumarate 3-hydroxylase; *C4H*, Coumarate-4-hydroxylase; *CAD*, Cinnamyl alcohol dehydrogenase; *CCR*, Cinnamoyl-CoA reductase; *CCoAOMT*, Caffeoyl-CoA *O*-methyltransferase; *COMT*, Caffeic acid/5-hydroxyconiferaldehyde O -methyltransferase; *F5H*, Ferulate 5-hydroxylase; *4CL*, 4-coumarate: CoA ligase; *HCT*, *p*-hydroxycinnamoyl-CoA:quinate shikimate *p*-hydroxycinnamoyltransferase.


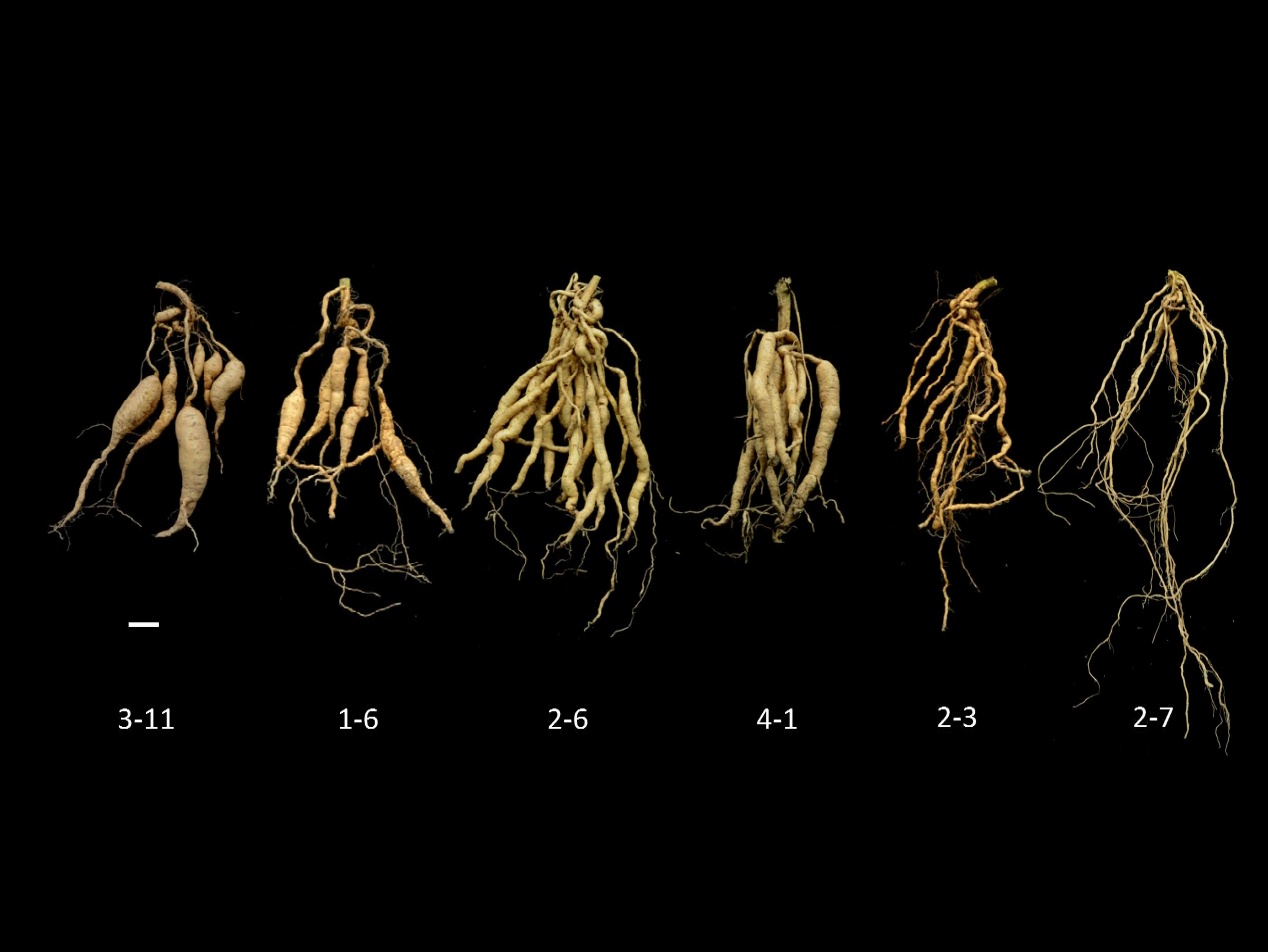


**Figure S17. Representative F1 individuals.**

3-11 had typical SR. 1-6, 2-6 and 4-1 had thickened roots (SR or SR-like; the xylem of some roots was partially lignified). 2-3 had pencil roots (PR). 2-7 had fibrous roots (FR). Scale bar: 2 cm.

**Figure S18. Frequency distribution of starch content in dry roots.**

13≤16 means that the starch content was higher than 13% and less than or equal to 16%; the ≤ symbol is used similarly throughout the x-axis labels.

**Figure S19. *Beta-amylase* gene numbers in five species.**


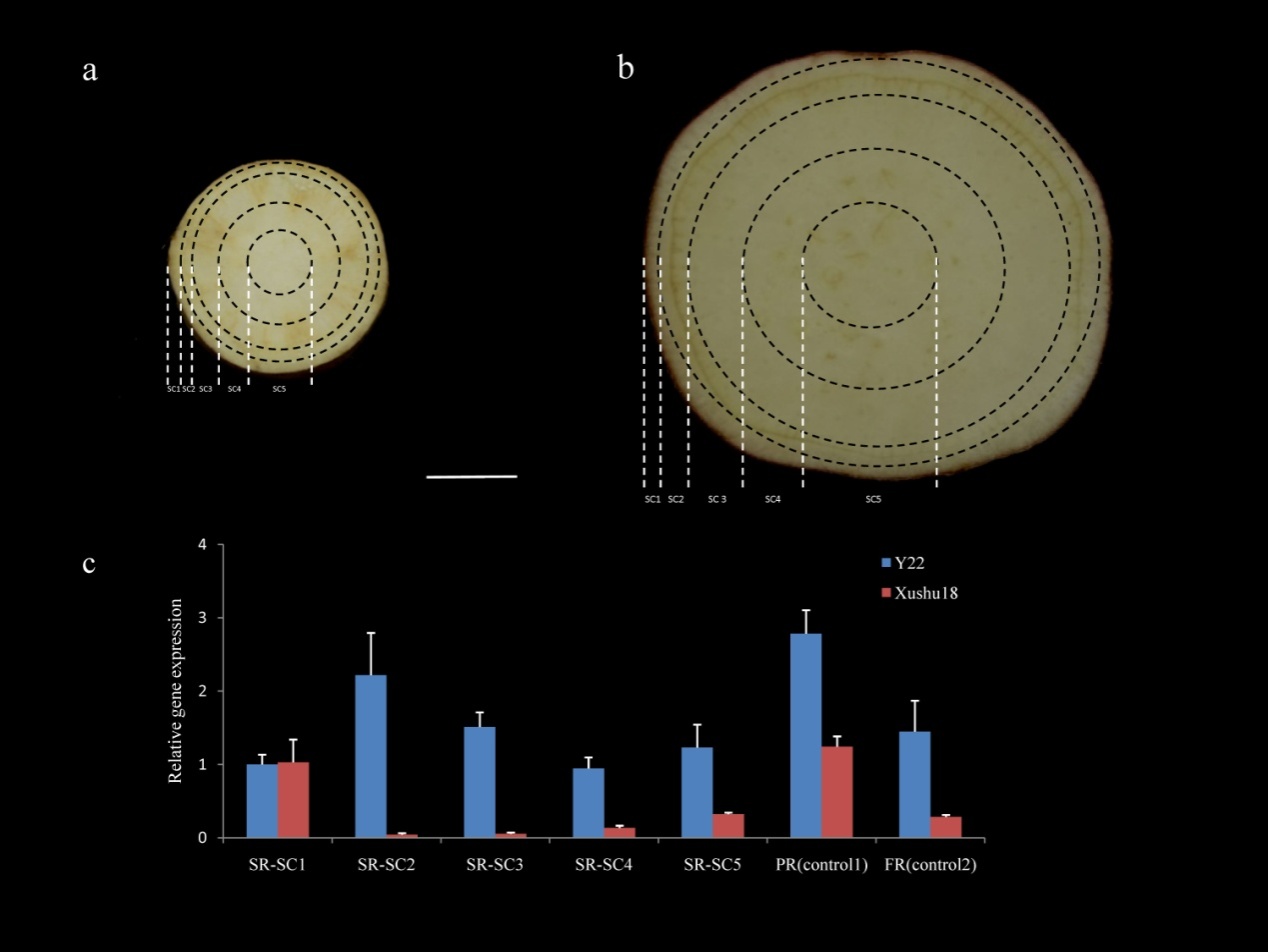


**Figure S20. Expression of *BMY11* in SR.**

**(a)** Transverse section of an SR from *I. trifida* var. Y22. The SR was sliced transversely and further divided into five sections: section 1 was the outer section of the cortex including the epidermis (SC1), section 2 included the inner section of the cortex and outermost portion of the xylem (SC2), section 3 was the outer part of the xylem (SC3), section 4 was the middle part of the xylem (SC4), and section 5 was the inner part of the xylem (SC5). PR and FR were used as controls. **(b)** Transverse section of an SR from sweetpotato var. Xushu18. The SR was sectioned as in (a). Bar=10 mm. **(c)** qRT-PCR results of *BMY11* in the transverse SR sections of Y22 and Xushu18.
