# Supplementary material for: The wild sweetpotato (Ipomoea trifida) genome provides insights into storage root development
Source: BMC Plant Biol. 2019 Apr 1;19:119. doi: 10.1186/s12870-019-1708-z (PMC6444543; doi:10.1186/s12870-019-1708-z)
Supplement: Supplementary file 2 — Table S1. Survey statistic results. Table S2. Sequencing data statistics. Table S3. Statistics of assembly results with only Illumina sequencing data. Table S4. Statistics of assembly results after extension with PacBio RS II data. Table S5. SSR primer used for identification of true F1 hybrids. Table S6. Statistical information of genetic linkage groups. Table S7. Coverage statistics of the I. trifida genome. Table S8. EST sequence evaluation results. Table S9. CEGMA evaluation results. Table S10. RNA-seq data used for annotation. Table S11. Statistical results of gene functional annotations. Table S12. Statistical results of non-coding RNAs. Table S13. Statistical results of repeated classification. Table S14. Summary of the plant species and assemblies/gene models used in this study. Table S15. Comparison of repeat contents between I. trifida and I. nil. Table S16. Statistics of LTR numbers. Table S17. Lengths of syntenic blocks and block repeat sequences. Table S18. Chromosome duplication test results for Y22. Table S19. KEGG enrichment results for the genes expanded in I. trifida. Table S20. KEGG enrichment results for the genes contracted in I. trifida. Table S21. Sequencing data statistics of RNA from S0 to S3. Table S22. KEGG pathways of upregulated genes. Table S23. Blast results of the specific protein sporamin in the I. trifida assembly. Table S24. KEGG pathways of downregulated genes. Table S25. QTL mapping results. Table S26. Results of BMY11 microarray hybridization during the process of SR development in sweetpotato var. Guangshu87. Table S27. Blast results of BMY11 in the full-length transcripts of sweetpotato var. Xushu18. Table S28. qPCR primers used to amplify BMY11. (DOCX 75 kb) [file 12870_2019_1708_MOESM2_ESM.docx]

**Additional file 2**

| **Table S1.** Survey statistic results. | | | | | |
| --- | --- | --- | --- | --- | --- |
| K-mer | K-mer number | K-mer depth | Genome size (Mbp) | Heterozygous ratio (%) | Repeat (%) |
| 17 | 30,979,252,564 | 65 | 476.6 | 2.20 | 48.42 |

| **Table S2.** Sequencing data statistics. | | | |
| --- | --- | --- | --- |
| Paired-end libraries | Insert size | Total data (G) | Sequence coverage (X)* |
| Illumina reads | 230 bp | 24.7 | 51.9 |
|  | 350 bp | 25.4 | 53.4 |
|  | 500 bp | 16.8 | 35.3 |
|  | 2 Kbp | 19.4 | 40.8 |
|  | 5 Kbp | 23.1 | 48.5 |
|  | 10 Kbp | 8 | 16.8 |
|  | 20 Kbp | 8.2 | 17.2 |
| Illumina Moleculo synthetic long reads | NA | 0.5 | 1.05 |
| PacBio long reads | NA | 10.05 | 21 |
| Total | - | 136.15 | 285.95 |
| * ~476 M genome size estimated by survey. | | | |

| **Table S3.** Statistics of assembly results with only Illumina sequencing data. | | | | |
| --- | --- | --- | --- | --- |
|  | Length | | Number | |
|  | Contig** (bp) | Scaffold (bp) | Contig** | Scaffold |
| Total | 404,918,082 | 431,572,764 | 28,234 | 5,422 |
| Max | 268,560 | 4,363,448 | - | - |
| Number>=2000 | - | - | 26,574 | 5,833 |
| N50 | 26,502 | 580,682 | 4,393 | 206 |
| N60 | 20,999 | 449,725 | 6,112 | 290 |
| N70 | 16,107 | 325,837 | 8,308 | 403 |
| N80 | 11,549 | 208,946 | 11,266 | 567 |
| N90 | 6,965 | 100,522 | 15,724 | 856 |
| ** Contig after scaffolding | | | | |

| **Table S4.** Statistics of assembly results after extension with PacBio RS II data. | | | | |
| --- | --- | --- | --- | --- |
| Sample ID | Length | | Number | |
|  | Contig** (bp) | Scaffold (bp) | Contig** | Scaffold |
| Total | 449,490,753 | 460,931,543 | 16,598 | 5,264 |
| Max | 554,601 | 4,534,310 | - | - |
| Number>=2000 | - | - | 14,426 | 3,620 |
| N50 | 54,490 | 607,924 | 2,372 | 211 |
| N60 | 43,084 | 471,328 | 3,301 | 297 |
| N70 | 33,168 | 343,488 | 4,481 | 412 |
| N80 | 23,648 | 217,240 | 6,075 | 580 |
| N90 | 14,248 | 98,949 | 8,488 | 884 |
| ** Contig after scaffolding | | | | |

| **Table S5.** SSR primer used for identification of true F1 hybrids. | |
| --- | --- |
| Forward | GCCTACGGGCTTGAAATGTA |
| Reverse | GTTGTTGGTCCCAAGTTGCT |

| **Table S6.** Statistical information of genetic linkage groups. | | | | | |
| --- | --- | --- | --- | --- | --- |
| LGname | Bin_num | SNP_num | Map length  (cM) | Average distance (cM) | Max. gap  (cM) |
| chr01 | 470 | 1158 | 136.9 | 0.29 | 4.15 |
| chr02 | 569 | 1536 | 364.32 | 0.64 | 23.56 |
| chr03 | 489 | 1212 | 279.76 | 0.57 | 15.76 |
| chr04 | 375 | 962 | 219.92 | 0.59 | 9.7 |
| chr05 | 558 | 1390 | 220.32 | 0.39 | 4.8 |
| chr06 | 449 | 1061 | 209.19 | 0.47 | 6.34 |
| chr07 | 424 | 930 | 212.02 | 0.5 | 14.67 |
| chr08 | 415 | 1040 | 221.26 | 0.53 | 6.48 |
| chr09 | 274 | 595 | 125.42 | 0.46 | 7.78 |
| chr10 | 309 | 746 | 163.68 | 0.53 | 12.73 |
| chr11 | 253 | 608 | 212.22 | 0.84 | 9.87 |
| chr12 | 369 | 863 | 161.12 | 0.44 | 7.12 |
| chr13 | 486 | 1168 | 252.33 | 0.52 | 21.96 |
| chr14 | 378 | 886 | 144.1 | 0.38 | 6.45 |
| chr15 | 488 | 1371 | 233.99 | 0.48 | 11.76 |
| Total | 6306 | 15526 | 3156.55 | 0.50 | - |

| **Table S7**. Coverage statistics of the *I. trifida* genome. | |
| --- | --- |
| - | Percentage |
| Mapping rate (%) | 95.2 |
| Coverage (%) | 99.58 |
| Coverage at least 4X (%) | 99.05 |
| Coverage at least 10X (%) | 98.06 |
| Coverage at least 20X (%) | 95.76 |
| Average sequence depth: The average depth of each base in the genome that is covered by the reads. Coverage: The proportion of the genome covered by the reads. | |

| **Table S8.** EST sequence evaluation results. | | | | | | | |
| --- | --- | --- | --- | --- | --- | --- | --- |
| Dataset | Number | Total length (bp) | Sequences covered by assembly (%) * | With >90% sequence in one scaffold | | With >50% sequence in one scaffold | |
|  |  |  |  | Number | Percent (%) | Number | Percent (%) |
| >0 bp | 52,639 | 43,034,105 | 99.54 | 48,853 | 92.81 | 52,010 | 98.8 |
| >200 bp | 52,639 | 43,034,105 | 99.54 | 48,853 | 92.81 | 52,010 | 98.8 |
| >500 bp | 23,525 | 34,279,942 | 99.87 | 21,579 | 91.73 | 23,365 | 99.32 |
| >1 Kbp | 13,408 | 27,163,683 | 99.95 | 12,103 | 90.27 | 13,328 | 99.4 |
| >2 Kbp | 5,113 | 15,234,736 | 99.94 | 4,434 | 86.72 | 5,070 | 99.16 |
| * Mapping ratio of EST sequences to genomic sequences. | | | | | | | |

| **Table** S**9.** CEGMA evaluation results. | | | |
| --- | --- | --- | --- |
| Complete | | Complete + partial | |
| # Prots | % Completeness | # Prots | % Completeness |
| 233 | 93.95 | 244 | 98.39 |
| Complete: core genes that were completely assembled with identity greater than 0.7.  Partial: core genes that were assembled but not complete.  # Prots: the number of core genes.  % Completeness: percentage of assembled core genes to core genes. | | | |

| **Table S10.** RNA-seq data used for annotation. | |
| --- | --- |
| Name | Total data (G) |
| Seed | 7.4 |
| Root | 5.4 |
| Stem | 4.2 |
| Leaf | 24.9 |
| Pollen | 20.8 |
| Stigma | 8.4 |
| Flower | 12.7 |

| **Table S11.** Statistical results of gene functional annotations. | | |
| --- | --- | --- |
|  | Number | Percent (%) |
| Total | 30,227 | - |
| InterPro | 23,190 | 76.72 |
| KEGG | 20,806 | 68.83 |
| Swiss-Prot | 23,365 | 77.30 |
| TrEMBL | 28,396 | 93.94 |
| GO | 17,004 | 56.25 |
| Annotated | 28,456 | 94.14 |
| Un-annotated | 1,924 | 5.86 |

| **Table S12.** Statistical results of non-coding RNAs. | | | | | |
| --- | --- | --- | --- | --- | --- |
| Type | | Copy | Average length (bp) | Total length (bp) | % of genome |
| miRNA | | 1,100 | 129.71 | 142,677 | 0.0310 |
| tRNA | | 2,595 | 75.19 | 195,108 | 0.0423 |
| rRNA | rRNA | 170 | 182.03 | 30,945 | 0.0067 |
|  | 18S | 113 | 216.96 | 24,517 | 0.0053 |
|  | 28S | 29 | 123.86 | 3,592 | 0.0008 |
|  | 5.8S | 17 | 115.41 | 1,962 | 0.0004 |
|  | 5S | 11 | 79.45 | 874 | 0.0002 |
| snRNA | snRNA | 661 | 122.08 | 80,695 | 0.0175 |
|  | CD-box | 316 | 101.19 | 31,976 | 0.0069 |
|  | HACA-box | 60 | 130.55 | 7,833 | 0.0017 |
|  | Splicing | 282 | 143.4 | 40,440 | 0.0088 |

| **Table S13.** Statistical results of repeated classification. | | | | | | |
| --- | --- | --- | --- | --- | --- | --- |
|  | Repbase TEs | | TE Proteins | | Combined TEs | |
|  | Length (bp) | % in Genome | Length (bp) | % in Genome | Length (bp) | % in Genome |
| DNA | 57,980,335 | 12.57 | 6,222,788 | 1.35 | 60,591,130 | 13.15 |
| LINE | 19,169,999 | 4.16 | 9,614,561 | 2.09 | 22,385,772 | 4.86 |
| SINE | 3,139,707 | 0.68 | 0 | 0 | 3,139,707 | 0.68 |
| LTR | 138,809,157 | 30.11 | 25,599,350 | 5.55 | 140,169,806 | 30.41 |
| Other | 23,443 | 0.01 | 0 | 0 | 23,443 | 0.01 |
| Unknown | 18,619,906 | 4.04 | 0 | 0 | 18,619,906 | 4.64 |
| Total | 221,743,436 | 48.11 | 41,377,415 | 8.79 | 226,047,594 | 49.04 |
| The results of this table do not contain TRF results (tandem repeats). Repbase TEs and TE proteins are transposable elements based on the Repbase library annotated by the RepeatMasker and RepeatProteinMask software, respectively. Combined TEs are the result of integrating the above three methods and removing redundancy. Other means that the repeat sequence can be classified by RepeatMasker, but it does not belong to the above categories. Unknown means that the repeat sequence cannot be classified by RepeatMasker. | | | | | | |

| **Table S14.** Summary of the plant species and assemblies/gene models used in this study. | | |
| --- | --- | --- |
| Species | Assembly/gene model | Version |
| *Arabidopsis thaliana* | http://plants.ensembl.org/Arabidopsis_thaliana | TAIR10.26 |
| *Manihot esculenta* | http://phytozome.jgi.doe.gov Manihot esculenta | v6.1 |
| *Solanum lycopersicum* | http://plants.ensembl.org/Solanum_lycopersicum | SL2.40.26 |
| *Solanum tuberosum* | http://plants.ensembl.org/Solanum_tuberosum | SolTub3.0.26 |
| *Ipomoea nil* | http://www.ddbj.nig.ac.jp/sub/wgs-e.html | Version 1.0 |
| *Oryza sativa* | http://plants.ensembl.org/Oryza_sativa/Info/Index | IRGSP-1.0.27 |
| *Coffea canephora* | http://coffee-genome.org | Version 1.0 |
| *Ipomoea trifida* | Our assembly |  |

| **Table S15.** Comparison of repeat contents between *I. trifida* and *I. nil.* | | |
| --- | --- | --- |
| Type | *I. trifida* | *I. nil* |
|  | % in genome | % in genome |
| DNA | 13.145364 | 9.770245 |
| LINE | 4.856637 | 3.891536 |
| SINE | 0.681166 | 0.718723 |
| LTR | 30.410114 | 38.992019 |
| Other | 0.005086 | 0 |
| Satellite | 0.040771 | 0.285198 |
| Simple_repeat | 0.461027 | 0.4423 |
| Unknown | 4.039625 | 0.91118 |
| Total | 49.041468 | 52.64155 |

| **Table S16.** Statistics of LTR numbers. | | | |
| --- | --- | --- | --- |
|  | solo_LTR | intact_LTR-RT | Ratio |
| *I. trifida* | 5044 | 5239 | 0.962779156 |
| *I. nil* | 7197 | 19080 | 0.377201258 |

| **Table S17.** Lengths of syntenic blocks and block repeat sequences. | | |  |
| --- | --- | --- | --- |
|  | Length of syntenic block | Length of syntenic block repeat sequences | Ratio |
| *I. trifida* | 248,323,054 | 97,100,003 | 0.391022909 |
| *I. nil* | 339,926,998 | 164,983,757 | 0.485350555 |

| **Table S18.** Chromosome duplication test results for Y22. | | | | |
| --- | --- | --- | --- | --- |
| Ratio | Y22:Vvi | | Y22:Cca | |
|  | Y22 coverage (%) | Vvi coverage (%) | Y22 coverage (%) | Cca coverage (%) |
| 1:1 | 63.6 | 94.2 | 55.3 | 94.5 |
| 2:1 | 88.4 | 95.8 | 83.8 | 95.2 |
| 3:1 | 93.2 | 95.8 | 93.2 | 95.4 |
| 4:1 | 93.2 | 95.8 | 93.2 | 95.4 |
| 6:1 | 93.2 | 95.8 | 93.2 | 95.4 |

Vvi: *Vitis vinifera.* Cca: *Coffea canephora.*

| **Table S19.** KEGG enrichment results for the genes expanded in *I. trifida* | | |
| --- | --- | --- |
| MapID | MapTitle | P-value |
| map00053 | Ascorbate and aldarate metabolism | 4.02E-16 |
| map00531 | Glycosaminoglycan degradation | 1.59E-09 |
| map00604 | Glycosphingolipid biosynthesis - ganglio series | 1.43E-08 |
| map00945 | Stilbenoid, diarylheptanoid and gingerol biosynthesis | 8.97E-08 |
| map00511 | Other glycan degradation | 1.08E-06 |
| map03020 | RNA polymerase | 2.16E-06 |
| map00600 | Sphingolipid metabolism | 1.36E-05 |
| map00240 | Pyrimidine metabolism | 3.83E-05 |
| map04144 | Endocytosis | 7.38E-05 |
| map02010 | ABC transporters | 5.85E-04 |
| map00903 | Limonene and pinene degradation | 8.11E-04 |
| map03018 | RNA degradation | 1.85E-03 |
| map04141 | Protein processing in endoplasmic reticulum | 4.53E-03 |
| map04146 | Peroxisome | 5.62E-03 |
| map01200 | Carbon metabolism | 8.89E-03 |
| map00909 | Sesquiterpenoid and triterpenoid biosynthesis | 1.11E-02 |
| map00710 | Carbon fixation in photosynthetic organisms | 2.08E-02 |
| map03410 | Base excision repair | 3.80E-02 |
| map00950 | Isoquinoline alkaloid biosynthesis | 3.83E-02 |
| map03040 | Spliceosome | 4.03E-02 |
| map03010 | Ribosome | 4.52E-02 |
| map04712 | Circadian rhythm - plant | 4.53E-02 |

| **Table S20.** KEGG enrichment results for the genes contracted in *I. trifida* | | |
| --- | --- | --- |
| MapID | MapTitle | P-value |
| map04626 | Plant-pathogen interaction | 3.19E-28 |
| map02010 | ABC transporters | 2.11E-10 |
| map00053 | Ascorbate and aldarate metabolism | 2.94E-07 |
| map00460 | Cyanoamino acid metabolism | 2.28E-06 |
| map00966 | Glucosinolate biosynthesis | 2.54E-06 |
| map00591 | Linoleic acid metabolism | 2.76E-06 |
| map00592 | alpha-Linolenic acid metabolism | 2.10E-05 |
| map00909 | Sesquiterpenoid and triterpenoid biosynthesis | 4.87E-05 |
| map00040 | Pentose and glucuronate interconversions | 1.29E-03 |
| map00270 | Cysteine and methionine metabolism | 5.60E-03 |
| map00902 | Monoterpenoid biosynthesis | 7.02E-03 |
| map00380 | Tryptophan metabolism | 7.55E-03 |
| map00240 | Pyrimidine metabolism | 1.64E-02 |
| map03040 | Spliceosome | 3.01E-02 |
| map00740 | Riboflavin metabolism | 3.25E-02 |

| **Table S21.** Sequencing data statistics of RNA from S0 to S3. | | | | |
| --- | --- | --- | --- | --- |
| **Sample name** | **Raw reads** | **Clean reads** | **Clean bases** | **Q20(%)** |
| S0_1 | 40164306 | 39,322,386 | 5.90G | 97.52 |
| S0_2 | 41741418 | 40,830,986 | 6.12G | 97.98 |
| S0_3 | 45277900 | 44,524,328 | 6.68G | 97.80 |
| S1_1 | 44042058 | 43,107,078 | 6.46G | 97.56 |
| S1_2 | 42184972 | 41,485,332 | 6.22G | 97.29 |
| S1_3 | 45634312 | 45,020,490 | 6.76G | 97.48 |
| S2_1 | 38692560 | 37,668,742 | 5.66G | 96.60 |
| S2_2 | 43634480 | 42,712,114 | 6.40G | 96.26 |
| S2_3 | 43150150 | 42,503,954 | 6.38G | 97.19 |
| S3_1 | 41509550 | 40,455,614 | 6.06G | 97.03 |
| S3_2 | 41915536 | 40,828,596 | 6.12G | 96.73 |
| S3_3 | 42516556 | 41,362,900 | 6.20G | 96.61 |

| **Table S22.** KEGG pathways of upregulated genes. | | | | | |
| --- | --- | --- | --- | --- | --- |
| S1 vs S0 | | S2 vs S0 | | S3 vs S0 | |
| pathway_term | p-value | pathway_term | p-value | pathway_term | p-value |
| Starch and sucrose metabolism | 0.006231337 | Starch and sucrose metabolism | 0.005065388 | Starch and sucrose metabolism | 0.00134178 |
| Fructose and mannose metabolism | 0.043613166 | Biosynthesis of secondary metabolites | 0.045300455 | Thiamine metabolism | 0.006743238 |
|  |  | Fructose and mannose metabolism | 0.045300455 | Terpenoid backbone biosynthesis | 0.012938874 |

| **Table S23.** Blast results of the specific protein sporamin in the *I. trifida* assembly. | | | | | | | | | | | | |
| --- | --- | --- | --- | --- | --- | --- | --- | --- | --- | --- | --- | --- |
| Query_id | Query  length | Query  start | Query  end | Q_align  ratio | Strand | Subject_id | Subject_  length | Subject  start | Subject_  end | S_align  ratio | Score | Identity |
| SPOR531 | 224 | 18 | 219 | 0.9 | + | Itr.xfSc0000003.219 | 219 | 18 | 219 | 0.92 | 194 | 96.04 |
| SPO_B1 | 221 | 1 | 216 | 0.98 | + | Itr.xfSc0000003.220 | 216 | 1 | 216 | 1 | 213 | 98.61 |

| **Table S24.** KEGG pathways of downregulated genes. | | | | | |
| --- | --- | --- | --- | --- | --- |
| S1 vs S0 | | S2 vs S0 | | S3 vs S0 | |
| pathway_term | P-value | pathway_term | P-value | pathway_term | P-value |
| Phenylalanine metabolism | 9.57E-13 | Phenylpropanoid biosynthesis | 3.45E-10 | Phenylpropanoid biosynthesis | 7.81E-15 |
| Phenylpropanoid biosynthesis | 1.49E-11 | Phenylalanine metabolism | 6.47E-08 | Phenylalanine metabolism | 2.41E-09 |
| Biosynthesis of secondary metabolites | 7.89E-05 | Biosynthesis of secondary metabolites | 1.12E-05 | Plant-pathogen interaction | 2.04E-07 |
| Stilbenoid, diarylheptanoid and gingerol biosynthesis | 0.001966246 | Stilbenoid, diarylheptanoid and gingerol biosynthesis | 1.65E-05 | Stilbenoid, diarylheptanoid and gingerol biosynthesis | 2.04E-06 |
| Flavonoid biosynthesis | 0.017957833 | Plant-pathogen interaction | 9.92E-05 | Biosynthesis of secondary metabolites | 6.26E-05 |
| Nicotinate and nicotinamide metabolism | 0.022812026 | Flavonoid biosynthesis | 0.000644403 | Flavonoid biosynthesis | 0.000221461 |
|  |  |  |  | Plant hormone signal transduction | 0.000738752 |
|  |  |  |  | Sesquiterpenoid and triterpenoid biosynthesis | 0.001919496 |
|  |  |  |  | Starch and sucrose metabolism | 0.027784126 |

| **Table S25.** QTL mapping results. | | | | | | | | |
| --- | --- | --- | --- | --- | --- | --- | --- | --- |
| Phenotype | Linkage group | Position | Locus | LOD | % Expl | Variance | Left marker | Right marker |
| Starch | lg03 | 104.50 | lm207 | 5.29 | 11.5 | 30.47 | lm205 | lm1915 |
| Starch | lg03 | 105.51 | lm209 | 5.88 | 12.7 | 30.05 | lm208 | lm211 |
| Starch | lg03 | 112.03 | lm3670 | 7.87 | 16.6 | 28.71 | lm2830 | hk286 |
| Starch | lg03 | 216.61 | np1545 | 5.92 | 12.7 | 30.03 | lm920 | lm1460 |
| Starch | lg03 | 217.28 | np73 | 7.78 | 16.4 | 28.77 | np682 | np804 |
| Expl (%): The ratio of phenotypic variation explained by the QTL. Variance: the residual variance after fitting the QTL. | | | | | | | | |

| **Table S26.** Results of *BMY11* microarray hybridization during the process of SR development in sweetpotato var. Guangshu87. | | | | | | | |
| --- | --- | --- | --- | --- | --- | --- | --- |
| DAT | 10 vs 10 | 15 vs 10 | 20 vs 10 | 30 vs 10 | 60 vs 10 | 90 vs 10 | 120 vs 10 |
| Relative expression | 1 | 1.38196 | 1.73457 | 1.97391 | 2.03417 | 1.87384 | 1.87810 |
| DAT: days after transplanting. For microarray analysis, root samples from 10 DAT, 15 DAT, 30 DAT, 60 DAT, 90 DAT and 120 DAT were used. ProbeSetID: IBTC1002867. Using the 10 DAT sample as a reference, the value of the log2 ratio was used to determine the differential expression levels between the developmental stages. | | | | | | | |

| **Table S27.** Blast results of *BMY11* in the full-length transcripts of sweetpotato var. Xushu18. | | | | | | | | | | | | |
| --- | --- | --- | --- | --- | --- | --- | --- | --- | --- | --- | --- | --- |
| #Query_id | Query_length | Query_start | Query_end | Q_align_ratio | Strand | Subject_id | Subject_length | Subject_start | Subject_end | S_align_ratio | Score | Identity |
| Itr.Sc0000035.86 | 1647 | 1 | 1647 | 1 | + | c28397_1_2182_Sam1_2-3k | 2182 | 179 | 1925 | 0.8 | 1638 | 99.45 |
| Itr.Sc0000035.86 | 1647 | 1 | 1647 | 1 | + | c29435_1_1908_Sam1_1-2k | 1908 | 35 | 1681 | 0.86 | 1636 | 99.27 |
| Itr.Sc0000035.86 | 1647 | 1 | 1647 | 1 | + | c4069_1_2059_Sam1_1-2k | 2059 | 157 | 1803 | 0.8 | 1636 | 99.15 |
| Itr.Sc0000035.86 | 1647 | 1 | 1647 | 1 | + | c32752_1_1931_Sam1_1-2k | 1931 | 33 | 1765 | 0.9 | 1634 | 99.21 |
| Itr.Sc0000035.86 | 1647 | 1 | 1647 | 1 | + | c30477_1_1899_Sam1_1-2k | 1899 | 156 | 1795 | 0.86 | 1621 | 98.12 |
| Itr.Sc0000035.86 | 1647 | 46 | 1646 | 0.97 | + | c35296_1_1848_Sam1_1-2k | 1848 | 69 | 1585 | 0.82 | 1071 | 89.25 |
| Itr.Sc0000035.86 | 1647 | 9 | 1643 | 0.99 | + | c6057_1_4025_Sam1_2-3k | 4025 | 34 | 3910 | 0.96 | 579 | 94.92 |
| Itr.Sc0000035.86 | 1647 | 362 | 401 | 0.02 | + | c39609_1_2726_Sam1_2-3k | 2726 | 596 | 635 | 0.01 | 36 | 90 |
| Itr.Sc0000035.86 | 1647 | 362 | 401 | 0.02 | + | c31944_1_1918_Sam1_1-2k | 1918 | 599 | 638 | 0.02 | 36 | 90 |
| Itr.Sc0000035.86 | 1647 | 362 | 401 | 0.02 | - | c23267_1_1953_Sam1_1-2k | 1953 | 1432 | 1471 | 0.02 | 36 | 90 |
| Itr.Sc0000035.86 | 1647 | 362 | 401 | 0.02 | + | c20186_1_2025_Sam1_1-2k | 2025 | 595 | 634 | 0.02 | 36 | 90 |
| Itr.Sc0000035.86 | 1647 | 362 | 401 | 0.02 | + | c8451_4_1777_Sam1_1-2k | 1777 | 346 | 385 | 0.02 | 36 | 90 |
| Itr.Sc0000035.86 | 1647 | 362 | 401 | 0.02 | + | c631_5_2063_Sam1_1-2k | 2063 | 603 | 642 | 0.02 | 36 | 90 |
| Itr.Sc0000035.86 | 1647 | 365 | 391 | 0.02 | - | 17514_Ib.all.nr.fas | 242 | 216 | 242 | 0.11 | 26 | 96.3 |
| Itr.Sc0000035.86 | 1647 | 293 | 318 | 0.02 | - | 27832_Ib.all.nr.fas | 477 | 452 | 477 | 0.05 | 25 | 96.15 |
| Itr.Sc0000035.86 | 1647 | 1508 | 1531 | 0.01 | + | c49661_1_4792_Sam1_3-6k | 4792 | 4681 | 4704 | 0.01 | 23 | 95.83 |
| Itr.Sc0000035.86 | 1647 | 292 | 311 | 0.01 | + | c25218_1_3757_Sam1_3-6k | 3757 | 394 | 413 | 0.01 | 20 | 100 |

| **Table S28.** qPCR primers used to amplify *BMY11*. | |
| --- | --- |
| Name | Primer sequence |
| actin_F | 5’-GGTGTTATGGTTGGGATGGGAC-3’ |
| actin_R | 5’-GGTAAGAAGGACAGGGTGCTC-3’ |
| BMY11-F | 5’-CCATCTCACCCATCAGAACTAAC-3’ |
| BMY11-R | 5’-TAAGATAACCTTCGGGACTGC-3’ |
